# Standardized Computer-based Organized Reporting of EEG: SCORE

**DOI:** 10.1111/epi.12135

**Published:** 2013-03-18

**Authors:** Sándor Beniczky, Harald Aurlien, Jan C Brøgger, Anders Fuglsang-Frederiksen, António Martins-da-Silva, Eugen Trinka, Gerhard Visser, Guido Rubboli, Helle Hjalgrim, Hermann Stefan, Ingmar Rosén, Jana Zarubova, Judith Dobesberger, Jørgen Alving, Kjeld V Andersen, Martin Fabricius, Mary D Atkins, Miri Neufeld, Perrine Plouin, Petr Marusic, Ronit Pressler, Ruta Mameniskiene, Rüdiger Hopfengärtner, Walter Emde Boas, Peter Wolf

**Affiliations:** * Department of Clinical Neurophysiology, Danish Epilepsy CentreDianalund, Denmark; † Department of Clinical Neurophysiology, University of AarhusAarhus, Denmark; ‡ Department of Neurology, Haukeland University HospitalBergen, Norway and Department of Clinical Medicine, University of BergenBergen, Norway; § Department of Neurological Disorders, Hospital Santo António/CHP and UMIB/ICBAS–University of PortoPorto, Portugal; ¶ Department of Neurology, Paracelsus Medical UniversitySalzburg, Austria; # Department of Clinical Neurophysiology, Epilepsy Institute in the Netherlands (SEIN), “Meer and Bosch”Heemstede, The Netherlands; ** IRCCS Institute of Neurological Sciences, Bellaria HospitalBologna, Italy; †† Epilepsy Center, Neurological Clinic, University Hospital ErlangenErlangen, Germany; ‡‡ Department of Clinical Neurophysiology, University of LundLund, Sweden; §§ Department of Neurology, Thomayer HospitalPrague, Czech Republic; ¶¶ Department of Clinical Neurophysiology, Glostrup Hospital, University of CopenhagenCopenhagen, Denmark; ## Nørmark HospitalIshøj, Denmark; *** EEG and Epilepsy Unit, Department of Neurology, Tel-Aviv Sourasky Medical Center, Tel-Aviv UniversityTel-Aviv, Israel; ††† Hopital Necker Enfants MaladesParis, France; ‡‡‡ Department of Neurology, 2nd Faculty of Medicine, Charles University in Prague, Motol University HospitalPrague, Czech Republic; §§§ Great Ormond Street Hospital for Children, NHS Foundation TrustLondon, United Kingdom; ¶¶¶ Department of Neurology, Vilnius University Hospital “Santariškių klinikos” Vilnius, Lithuania and, Clinic of Neurology and Neurosurgery, Faculty of Medicine, Vilnius UniversityVilnius, Lithuania; #### Department of Neurology, Danish Epilepsy CentreDianalund, Denmark

**Keywords:** Assessment, Database, Definitions, EEG, Semiology, Terms

## Abstract

The electroencephalography (EEG) signal has a high complexity, and the process of extracting clinically relevant features is achieved by visual analysis of the recordings. The interobserver agreement in EEG interpretation is only moderate. This is partly due to the method of reporting the findings in free-text format. The purpose of our endeavor was to create a computer-based system for EEG assessment and reporting, where the physicians would construct the reports by choosing from predefined elements for each relevant EEG feature, as well as the clinical phenomena (for video-EEG recordings). A working group of EEG experts took part in consensus workshops in Dianalund, Denmark, in 2010 and 2011. The faculty was approved by the Commission on European Affairs of the International League Against Epilepsy (ILAE). The working group produced a consensus proposal that went through a pan-European review process, organized by the European Chapter of the International Federation of Clinical Neurophysiology. The Standardised Computer-based Organised Reporting of EEG (SCORE) software was constructed based on the terms and features of the consensus statement and it was tested in the clinical practice. The main elements of SCORE are the following: personal data of the patient, referral data, recording conditions, modulators, background activity, drowsiness and sleep, interictal findings, “episodes” (clinical or subclinical events), physiologic patterns, patterns of uncertain significance, artifacts, polygraphic channels, and diagnostic significance. The following specific aspects of the neonatal EEGs are scored: alertness, temporal organization, and spatial organization. For each EEG finding, relevant features are scored using predefined terms. Definitions are provided for all EEG terms and features. SCORE can potentially improve the quality of EEG assessment and reporting; it will help incorporate the results of computer-assisted analysis into the report, it will make possible the build-up of a multinational database, and it will help in training young neurophysiologists.

The interobserver agreement in electroencephalography (EEG) interpretation is only moderate (Van Donselaar et al., 1992; Stroink et al., 2006). The EEG signal has a high complexity. It depends on the intricate interplay between the activation of neural networks, localization and orientation (Wong, 1998) of the source (dipole), and its propagation throughout the brain (Lopes da Silva & van Rotterdam, 1993; Scherg et al., 1999; Flemming et al., 2005). Although certain aspects (such as spike detection) can be automated, the whole process of extracting clinically relevant features cannot be computerized: it requires the assessment and interpretation of the recording by trained experts. Therefore it is, to some extent, subjective and dependent on the abilities, training, and experience of the EEG reader (EEGer; Stubbe et al., 1982). Another factor probably contributing to the interobserver differences is the free-text format of the EEG descriptions. A recent study has demonstrated that the interobserver agreement was consistently higher when the observers had to choose mandatory terms than when they could use optional ones (Gerber et al., 2008). When assessed for a relatively well-defined and restricted feature (such as presence or absence of epileptiform discharges) the interobserver agreement has proved to be beyond 80% (Stroink et al., 2006). However, the EEG recordings are much more complex than to be described by a list of binominal measures. Free-text formats are sufficiently flexible to reflect the various features extracted and weighted by the observers, but it can also lead to suboptimal assessment of the recordings: it does not prevent the observer from missing important features, it allows the use of terms not widely accepted (local terminologies), and makes it difficult to transfer the results of EEG assessment from one laboratory to another (for building of a multinational database).

The objective of our endeavor was to construct software for characterizing EEG and ictal clinical events, where the physician chooses from predefined terms, simultaneously generating a report and filling information into a database. We wanted to develop a reliable tool to improve the quality of EEG assessment, to increase interobserver agreement in reporting EEG findings, to promote exchange of knowledge between EEG centers, to construct a multinational database for further research projects, and to assist in education and training.

There have been several successful attempts in medicine to standardize the evaluation and reporting of complex clinical and/or electrographic patterns, for example, the Unified Parkinson's Disease Rating Scale and the scoring of the polysomnography recordings using the American Academy of Sleep Medicine (AASM) Manual for the Scoring of Sleep and Associated Events.

Previous computerized EEG reporting systems have been published (Aurlien et al., 1999; Finnerup et al., 1999). Robert R. Young, Keith H. Chiappa, and their colleagues at Massachusetts General Hospital have in the 1980s used a locally developed software package for reporting EEG findings. Fixed terms could be selected and a report was semi-automatically generated (personal communication). Ronald Lesser and his colleagues at Johns Hopkins have been using a locally developed software package (“Reporter”) since 1998 for reporting EEG findings. Since then they have prepared 38,000 reports using the software (personal communication). Although “Reporter” does not construct a formal database, terms in the EEG descriptions are standardized, so that “keyword” searches can be used to help with a variety of needs, including construction of a database. However, the previous attempts did not reach broad international acceptance. One of them seemed to be too time-consuming in the clinical practice (Finnerup et al., 1999) and it only built a database, but not a report. Harald Aurlien and his colleagues have been using software (“Holberg”) for reporting EEG findings and generating a database since 1998, and a modified version of this software has been used for reporting all standard EEG reports at the Danish Epilepsy Centre since 2009. In total, >36,000 EEG studies have been reported using this software. The database that was automatically generated during the reporting made possible to address specific issues related to certain aspects of the EEG, and this led to three additional publications (Aurlien et al., 2004, 2007, 2009). However, these software packages remained in local use only. Probably the reasons for failing to reach a wider acceptance were the very different needs and traditions in different countries. To circumvent these problems, we tried to make the software as user-friendly as possible, and we tried to reach an international (in the first step a European) consensus on the structure and terms necessary for interpreting and reporting the EEG.

## Methods

A working group of EEG experts took part in consensus workshops in Dianalund, Denmark in 2010 and 2011. The faculty of the workshop was approved by the Commission on European Affairs (CEA) of the International League Against Epilepsy (ILAE), and the event was advertised on the homepage of the European Epilepsy Academy, the education and research organization of the CEA-ILAE. The SCORE working group, consisting of 25 clinical neurophysiologists/epileptologists from 15 European countries, elaborated a consensus proposal meant to reflect the needs and practice in different countries/centers. This consensus proposal was subsequently submitted to a pan-European review, organized by the European Chapter of the International Federation of Clinical Neurophysiology (IFCN).

The SCORE working group followed the widely accepted international standards: we incorporated the available, relevant guidelines, consensus statements, and task force proposals (Chatrian et al., 1974; Committee, 1981; Daube et al., 1993; Glimore, 1994; Noachtar et al., 1999; Blume et al., 2001; Flink et al., 2002; AASM, 2007; ACNS, 2007) as well as the terms described in authoritative EEG textbooks (Ebersole & Pedley, 2003; Niedermeyer & da Silva, 2005). We added to them or modified them only when absolutely necessary, based on published evidence and/or the consensus of the group. Because video-EEG recordings contain data on seizure semiology too, we attempted to include this into the structured report. The definitions for the terms used in SCORE are attached as Supporting Information. In the software these definitions are directly available for each term.

Based on the consensus statement, the SCORE software has been developed by a group of programmers at the Holberg EEG AS. The programming work was organized and supervised by one of the authors (HA), and, during this process, the content of the software was repeatedly compared and synchronized with the consensus statement by another author (SB).

The development of the software took 3 years. It was an iterative process, where successive versions were tested by scoring EEG recordings in the clinical practice, and the software was continuously corrected and adjusted. In total, >2,000 recordings were included in this process, and four major revisions were made, in addition to the numerous corrections and trouble-shooting.

A free version of the SCORE software and a detailed guidance for users can be requested from the following home-page: http://holbergeeg.com.

The software automatically generates a report and saves the scored features in a local database.

## Results

Table 1 shows the main elements of SCORE, constituting the flowchart of data evaluation and interpretation.

**Table 1:** The main elements of SCORE: the flowchart

1. The patient's personal data
2. Referral data
3. Recording conditions
4. Modulators/procedures
5. Background activity
6. Sleep and drowsiness
7. Interictal findings
8. Episodes
9. Physiologic patterns
10. Patterns of uncertain significance
11. EEG artifacts
12. Polygraphic channels
13. Diagnostic significance

Elements 1–4 can be filled in by the EEG technician (physiologist), and later checked by the physician who interprets the recording. In the future, connecting the SCORE software with the patient administrative system of the hospital/EEG department would help filling in these administrative data. Naturally, several recordings can be listed for each patient. Each recording has its own referral.

Elements 5–12 contain the main features assessed during the process of reading the EEG recordings (grouped in the software under “findings”). The list is long, because it is meant to contain all clinically relevant aspects that can occur during a diagnostic EEG recording. However, the software was designed in such a way that the user does not have to waste time on those features that do not occur in the recording. In other words, if an element/feature is not applicable for the recording to be described, one should not open it from the list. The user chooses the complexity of scoring according to the clinical setting and the recording.

The last element contains the overall interpretation of the recording, where the physician specifies the diagnostic significance. When this is done, a report is automatically generated, and the features scored by the user are fed into the database. The terms in the main flowchart are defined in Appendix S1.

### Personal data, referral, and recording conditions

SCORE is installed and run within the hospitals' information technology system. The users must ensure that the software is used according to the local regulations for security of personal data. An “anonymization” function will be available. This will remove all personal data from an entry and keep only the scored EEG features.

*The patient's personal data* (“patient details”) contain obligatory elements and optional ones. The obligatory elements are the following: identity number (“identity string” – in most countries this is given by the social security number), last name, first name, and date of birth. Optionally one can record the patient's address and other details (under the entry: “notes”). For patients younger than 3 months, the option of recording the mother's name instead of the first name of the patient is offered. For patients younger than 12 months, registering the gestational age is offered as an option. In the next step, the recording conditions and the referral data are entered.

If the patient is younger than 3 weeks at the time of the recording, the software automatically switches to the special, neonatal template; for patients between 3 and 5 weeks of age, the physician can opt to use (or not) the neonatal reporting-matrix.

*The recording conditions* contain: start time, duration of the recording, EEG type (standard/sleep deprived/ambulatory recording/short-term video-EEG recording/long-term video-EEG monitoring/recording in the intensive care unit), and sensor group (10-20 system, 10-10 system). The sensor array can be customized for each user site, in the settings. The name of the technician, physician, and supervision physician (if any) is selected here. The alertness of the patient is also registered by selecting item(s) from a multiple-choice list: awake/oriented/good cooperation/poor cooperation/disoriented/drowsy/asleep/stupor/comatose. Optionally the time of the patient's latest meal can be added. Other aspects considered important for the recording can be detailed under “notes.”

*The* “*referral*” *part* contains the following entries: referring unit, reason(s) for referral, diagnosis at referral (International Classification of Diseases, 10th Edition [ICD-10] list), latest seizure, medication (from the World Health Organization [WHO] list, with the possibility of specifying also medication withdrawal and medication administered during the recording). A list of choices is offered for the reasons for referral: epilepsy-related indications, other differential diagnostic questions, specific pediatric indications, follow-up EEG, assessment of prognosis, and other indications (Table 2). Several, clinically relevant, non-EEG data can be entered here.

**Table 2:** Reasons for referral

Epilepsy-related indications
Clinical suspicion of epilepsy or seizure
Reconsider the initial diagnosis of epilepsy
Classification of a patient diagnosed with epilepsy
Changes in seizure pattern
Suspicion of nonconvulsive status epilepticus
Monitoring of status epilepticus
Monitoring of seizure frequency
Monitoring the effect of medication
Considering stopping AED therapy
Presurgical evaluation
Driver's license or flight certificate
Other differential diagnostic questions
Psychogenic nonepileptic seizures
Loss of consciousness
Disturbance of consciousness
Encephalopathy
Encephalitis
Dementia
Cerebral vascular disease
Paroxysmal behavioral changes
Other psychiatric
Behavioral symptoms
Coma
Brain death
Specific pediatric indications
Genetic syndrome
Metabolic disorder
Regression
Developmental problems
Follow-up EEG
Assessment of prognosis
Other indication

The options for the “latest seizure” entry are the following: “undetermined/unknown/<20 min/<1 h/<1 day/<1 week/<1 month/≥1 month.”

Basic information on brain magnetic resonance imaging (MRI) (normal/abnormal/not done/no data), functional neuroimaging tests (normal/abnormal/not done/no data), and cognitive impairment (no/mental retardation/dementia due to unique brain injury/dementia due to progressive disease) can be included. More detailed information on these aspects can be added as free text.

*The list of provocation methods* (“modulators/procedures”) performed during the recording conceptually belongs to the part with recording conditions. However, for technical reasons (programming) this is listed in the software as the first element of “findings.” This list contains the following: intermittent photic stimulation (IPS), hyperventilation, sleep deprivation, sleep (induced/natural/after sleep deprivation), awakening, medication administered during the recording, manual eye closure/opening, auditory stimulation, nociceptive stimulation, physical effort, cognitive tasks, and “other modulators and procedures” (specified in free text). Choosing hyperventilation prompts to the scoring of the quality of performance during this (insufficient or sufficient).

Until this point the data can be entered by the technicians/physiologist.

### Scoring the EEG

**Figure 1:**
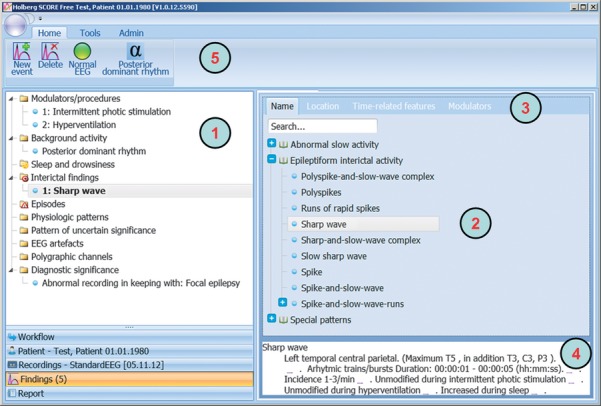
The interactive screen for scoring EEG studies. The upper left panel (1) contains the flowchart—the main elements to be scored. If an element is not relevant for the recording, it is simply skipped (it does not impose any additional burden). The selection panel (2) contains the features to be scored for the element that was chosen in the flowchart (in this case an interictal finding) and the corresponding choices. The upper row of tabs (3) in this panel shows the features available for scoring. In this example, for interictal findings the available features are: name, location, time-related feature, and modulators. When the “name” feature is active, the selection panel below displays the list of interictal graphoelements. After choosing “sharp-waves” from the name-list, the software automatically shifts to the next feature to be scored (in this example: location). The right lower panel (4) contains a summary of the scored items corresponding to the EEG finding that is being scored. The ribbon at the top (5) contains short-key functions (for example selecting “α” opens directly “Posterior dominant rhythm” with the selection box where frequency values are typed in).

Elements 5–12 are grouped under the heading “findings” in the SCORE software (Table 1). While reading the EEG, the physician “scores” the relevant features of the recordings using these entries. It follows the way electroencephalographers describe the EEG recordings. The first two elements contain the features of the “ongoing” EEG activity during wake period (“background activity”) and during drowsiness and sleep. Interictal findings depict all the graphoelements/EEG patterns that are considered abnormal, and that are not part of the ongoing (background) activity, and that are not the EEG manifestation of a seizure/clinical episode or of the postictal period; the presence of interictal discharges does not necessarily imply that the patient has epilepsy. The element “episodes” contains the clinical and EEG features of the seizures and other clinical/ictal events. Patterns of uncertain significance, physiologic patterns, artifacts, and polygraphic channels can also be scored, if relevant/applicable. In the end, the electroencephalographer scores the global interpretation/diagnostic significance of the recording. Finally a report is automatically generated. Figure 1 shows the interactive screen of the SCORE software.

### Background activity

Background activity contains three main subchapters: posterior dominant rhythm, other organized rhythms, and special features. The definition of the terms is detailed in Appendix S2.

Table 3 shows the features (in bold) that can be scored for *the posterior dominant rhythm* (alpha rhythm in adults) and the corresponding choices for each of them. For each recording the posterior dominant activity only can be scored only once. The electroencephalographer can score here the global interpretation of the posterior dominant activity for the patient (taking into account the age and the state of consciousness). The following choices are available: normal, abnormal, no definite abnormality, not possible to determine. To speed up scoring of most recordings, a short-key redirects the flowchart to the window where frequency of the posterior dominant rhythm can be specified.

**Table 3:** The features and choices for the posterior dominant rhythm

Frequency (Hz)	Amplitude (μV)	Reactivity to eye opening
Range (from… to …)	<20	Yes
or	20–50	Reduced – bilaterally
Single value (Hz)	50–100	Reduced on the left side
	100–200	Reduced on the right side
	>200	Not possible to determine
	Not possible to determine	

Amplitude asymmetry	Frequency asymmetry	Caveat
Symmetrical	Symmetrical	Only open eyes during the recording
L < R, abnormal left	No. Hz lower on the left side	Patient sleep deprived
L < R, abnormal right	No. Hz lower on the right side	Drowsy
R < L, abnormal left		
R < L, abnormal right		
Not possible to determine		

Absent posterior dominant rhythm because of
Artifacts
Extreme low voltage
Eye closure could not be achieved
Lack of awake period
Lack of compliance
Other (free text)

*Other organized rhythms* (besides the posterior dominant rhythm) can be selected. Here several entries are permitted (i.e., several types of “other organized rhythms” can be selected and successively scored for each recording). First, one has to choose a name of the rhythm: alpha, beta, mu, delta, theta. Then the localization has to be specified (see below). The extent (i.e., percentage of occurrence during the recording) and the reactivity to external stimuli (yes/no) can be selected. The electroencephalographer can score here the interpretation of this rhythm (taking into account the age and the state of consciousness) as normal, abnormal, no definite abnormality.

The last subchapter for the background activity is: “*special features*.” This contains three entities: electrocerebral inactivity, generalized suppression-burst, and suppression of the background activity. Selecting “electrocerebral inactivity/silence” makes it impossible to score any additional feature, except for “artifacts” and “diagnostic significance.” Selecting “generalized suppression-burst” prompts to the scoring of the duration of the bursts and (separately) the duration of the suppression. Selecting “suppression” prompts to the scoring of the duration of this. In the next step one can specify whether these entities are modulated (influenced) by external stimuli/interventions: passive eye opening, auditory stimuli, administered medication. The possible choices are: increase, decrease, unmodified, triggered by, stopped by, and “not possible to determine.”

### Scoring the location

Various entries (EEG patterns, graphoelements) have “location” as an attribute. This is described by laterality (left/right/midline/bilateral/diffuse) and regions (frontal, temporal, central, parietal, occipital). Scoring “laterality” and “regions” prompts (based on the sensor-settings specified in the recording conditions) a list with the sensors corresponding to the selected side and region. Form this list, individual electrodes can be unselected, and the maximum of the field potential can be specified (by choosing one or more electrode names).

For bilateral localizations, additional two features, the amplitude asymmetry, and the bilateral synchrony have to be scored. The choices for amplitude asymmetry are the following: symmetrical, consistently more pronounced on the left side (>50% difference), consistently more pronounced on the right side (>50% difference), shifting side-preponderance, not possible to determine). The choices for synchrony are the following: asynchronous, primary bilateral synchrony, secondary bilateral synchrony (propagation from left to right/from right to left), and cannot be determined.

The so-called “generalized” discharges are scored by choosing: “bilateral” + “synchronous” + name of the region.

“Diffuse” denotes a location of an EEG pattern (rhythm) that occurs in all or most of the regions, on both sides, but asynchronously.

If a graphoelement (transient) is seen in more than two locations, unrelated to each other (i.e., not part of the same discharge), “multifocal” can be selected as a descriptor of localization.

If propagation is seen within a graphoelement, this is scored in an additional location-window that is automatically added when choosing “propagation.”

Traditionally, the location of the EEG patterns/graphoelements is described by specifying the scalp regions (or electrodes) where the negative potentials are recorded. However, by visual assessment of the distribution of the negative and positive potentials on the scalp (voltage map) the brain region containing the source can be estimated. Optionally this can be registered as a location-descriptor. Choices for “brain regions” (and subregions in the brackets) are the following: frontal (perisylvian-superior surface; lateral; mesial; polar; orbitofrontal), temporal (polar; basal, lateral-anterior; lateral-posterior; perisylvian-inferior surface), central (lateral convexity; mesial; fissural-anterior, fissural-posterior; opercular), parietal (lateral-convexity; mesial; opercular), occipital (lateral; mesial), and insula.

### Sleep and drowsiness

The features of the “ongoing” activity during sleep are scored just following the “background activity.” If abnormal graphoelements (interictal findings) appear, disappear, or change their morphology during sleep, that is not scored here but at the entry corresponding to that graphoelement (under modulator/sleep). Giving a detailed description of sleep such as in polysomnography recordings is not the scope of this SCORE element. However, the features considered clinically relevant in an EEG recording are listed. The following entries can be selected: sleep architecture, normal sleep patterns, hypnagogic or hypnopompic hypersynchrony in children, sleep-onset rapid eye movement sleep (SOREM), abnormal asymmetry or absence of sleep graphoelements, nonreactive sleep activity. Appendix S3 contains the definitions of these terms.

Sleep architecture can be scored as normal/abnormal/not possible to determine. For “normal sleep patterns” the sleep stages reached during the recording can be specified (N1; N2; N3; rapid eye movement [REM]; not possible to determine). For the absence of sleep graphoelements, the name of the graphoelement (sleep spindles/vertex sharp transients/K-complex/positive occipital sharp transients of sleep/other) and the location where the graphoelement is absent or reduced (see scoring the localization) is specified. Significant asymmetry of the sleep spindles can be registered and the location (where it is reduced) can be specified. Several successive entries can be selected and scored for “sleep.”

### Interictal findings

Each interictal finding is characterized by four attributes (“features”): name of the graphoelement, localization, time-related features, and modulators (if any).

*The names* of the interictal graphoelements are listed in Table 4 and defined in Appendix S4. They are classified into four groups to make it easier to find the name in the list.

**Table 4:** Names of the interictal graphoelements

Epileptiform discharges	Special patterns
Polyspikes	Bi-PLEDs
Polyspike-and-slow-waves	Bursts suppression pattern
Runs of rapid spikes	Hypsarrhythmia
Sharp wave	PLEDs
Sharp-and-slow-waves	Periodic complex discharges – other than PLEDs
Slow sharp wave	
Spike	SIRPIDs
Spike-and-slow-wave	Triphasic waves
Spike-and-slow-wave runs	
Classical 3/s	
Slow 1–2.5/s	
Fast 4–5/s	

Abnormal slow activity	Neonatal
Delta	Alpha bursts
Polymorphic delta	Brief interictal rhythmic discharges (BIRDS)
Theta	
Delta and theta	Positive Rolandic sharp waves (PRSW)
Intermittent rhythmic slow activity	Positive temporal sharp-waves (PTS)
Frontal intermittent rhythmic delta activity (FIRDA)	
Occipital intermittent rhythmic delta activity (OIRDA)	
Temporal intermittent rhythmic delta activity (TIRDA)	

Bi-PLEDs, bilateral periodic lateralized epileptiform discharges; PLEDs, periodic lateralized epileptiform discharges; SIRPIDs, stimulus-induced rhythmic, periodic, or ictal discharges.

*The location* is scored as described above.

*The time-related features* of the interictal findings are discharge pattern, mode of appearance, and incidence.

“*Discharge pattern*” characterizes the time-related features within the discharge. The choices are: single discharges, rhythmic trains/bursts, and arrhythmic trains/bursts. The frequency is specified for the rhythmic bursts by entering the corresponding numbers (Hz). The duration is specified for the trains/bursts. Because some graphoelements might have more than one type of discharge pattern within a recording, multiple choices are allowed here.

“*Mode of appearance*” depicts how the interictal EEG patterns/graphoelements are distributed throughout the recording. The choices are: random, periodical, variable, and not possible to determine. If “periodical” is selected, the duration of the interdischarge interval can be specified.

“*Incidence*” characterizes how often the described interictal finding is seen throughout the recording. For the single-discharges, the suggested choices for “incidence” are: only once, <1 min, 1–3 min, 4–6 min, >1/10 s, and continuous. For the trains/bursts the incidence is expressed as the estimated percentage of the total duration of the bursts during the recording (<1%, 1–10%, 10–50%, 50–90%, >90%).

#### Modulators

Some interictal findings are influenced by external stimuli/interventions. These can be described as the fourth feature: “modulators.” The choices for eye-closure sensitivity are: yes and no. For the IPS the choices for photoparoxysmal response are: posterior stimulus-dependent response, posterior stimulus independent response (limited to the stimulus-train/sustained), and generalized photoparoxysmal response (limited to the stimulus-train/sustained), as suggested by Kasteleijn-Nolst Trenité et al. (2001). In addition, “activation of preexisting epileptogenic area” can be selected as a choice for IPS (Kasteleijn-Nolst Trenité et al., 2001; Beniczky et al., 2012). For the other modulators the following choices are available in SCORE: increase, decrease, unmodified, only during this modulator, not possible to determine. For “sleep” two additional choices are available: “change of pattern during sleep”—to make it possible to describe qualitative changes too (besides the quantitative changes described above), and “continuous during NREM sleep” to score for continuous spikes and waves during slow sleep (CSWS)/electrical status epilepticus in sleep (ESES); the percent is typed in the free-text box. Several, distinct interictal findings can be scored independently/successively.

### Episodes

This element of SCORE contains the descriptors of the clinical episodes and of electrographic seizures.

The main parts of this element are: name of the clinical episode, timing and context, effect of interventions, and electroclinical findings.

*The names* of the episodes can be selected from the list showed in Table 5 and Appendix S5. The table includes ILAE seizure classification (Commission, 1981; Berg et al., 2010). Focal seizures can be further classified according to the presumed localization, and it can be specified whether they evolve to bilateral convulsive seizure (impairment of consciousness is scored in the next step).

**Table 5:** Names of episodes

Generalized epileptic seizure
Tonic–clonic (in any combination)
Absence
Typical
Atypical
Absence with special features
Myoclonic absence
Eyelid myoclonia
Myoclonic
Myoclonic
Myoclonic atonic
Myoclonic tonic
Clonic
Tonic
Atonic
Focal seizure
Localization
Frontal/temporal/rolandic/parietal/occipital
Evolving to bilateral convulsive seizure
Other seizure types
Epileptic spasm
Tonic spasm
Unknown
Subtle seizure
Electrographic seizure
Psychogenic nonepileptic seizure (PNES)
Sleep-related events
Benign sleep myoclonus
Confusional awakening
Periodic limb movement in sleep (PLMS)
REM sleep behavioral disorder (RBD)
Sleepwalking
Pediatric events
Hyperekplexia
Jactatio capitis nocturna
Pavor nocturnus
Stereotypical behavior
Paroxysmal motor event
Other episodes
Syncope
Cataplexy
Other (free text)
Not possible to determine

“*Timing and context*” covers the following features: incidence (number of episodes/recording), time at start, duration of the episode and of the postictal phase, prodrome, state of wakefulness at the seizure start, impairment of consciousness during the seizure, provocative factors, facilitating factors, tongue biting, effect of medication, and time relationship between clinical and EEG start.

To reflect the clinical practice, SCORE makes it possible to group and describe several clinical episodes (seizures) under the same heading, if the physician considers them as manifestation of the same phenomenon. However, as a minimum, the seizure onset must be identical in all the clinical episodes described under the same heading. If several clinical episodes are described together, the number of such episodes during the recording and the time of their start have to be documented. For the cases where the precise number of the clinical events cannot be determined, this is included as a choice (“not possible to determine”).

The duration of the clinical episode (seconds) is registered. If several clinical events are described under the same heading, the range is registered (one enters two numbers). The option “>30 min” is given, if the precise length cannot be determined.

The prodrome (if any) can be selected, and then described in free text. Prodrome is a preictal phenomenon, and it is defined as a subjective or objective clinical alteration (e.g., ill-localized sensation or agitation) that heralds the onset of an epileptic seizure but does not form part of it (Blume et al., 2001). Therefore, prodrome should be distinguished from aura (which is an ictal phenomenon).

One can specify whether the clinical event started from sleep or from wake state. The impairment of consciousness during the seizure (affected/mildly affected/not affected/not possible to determine) can be scored here.

The facilitating factors (if known) can be selected: alcohol, awakening, catamenial, fever, sleep, sleep-deprivation, other (free text). Facilitating factors are defined as transient and sporadic endogenous or exogenous elements capable of augmenting seizure incidence (increasing the likelihood of seizure occurrence). The provocative factors (if known) can be selected from the list: hyperventilation, reflex (+free text), other (+free text). For IPS a list is offered to select the type of photoparoxysmal response (see under: “Modulators”). Provocative factors are defined as transient and sporadic endogenous or exogenous elements capable of evoking/triggering seizures immediately following the exposure to it.

Tongue biting can be selected and registered.

In this part one can specify whether the clinical start precedes the EEG start or the other way around. The time (in seconds) between the clinical and EEG start can be documented by entering the corresponding number.

#### Effect of interventions

If medication was administered during the clinical event (for example, to stop an epileptic seizure) the effect of medication can be scored: clinical effect (yes/no/not possible to determine) and the EEG changes (decrease/cessation/no change/increase/not possible to determine). Duration of the changes induced by medication administered during the recording can be entered here.

*The electroclinical findings* (i.e., the seizure semiology and the ictal EEG) are divided in three phases: onset, propagation, and postictal. For simple/short seizures the whole seizure can be described under “onset.”

Within the onset period several clinical signs can be registered, but this implies that they occurred simultaneously. For the propagation phase, several clinical signs/ictal EEG patterns can be selected, and their chronologic order of appearance can be specified. Because the elements of the propagation might vary within the group of clinical episodes described under the same heading, the number of episodes in which that particular element occurred can be specified. Otherwise the scoring of the onset and propagation phase is identical.

*The clinical signs* are described by a name and the body localization where it is observed. The list with the names (Table 6, Appendix S5) corresponds to the ILAE Commission Report: Glossary of Descriptive Terminology for Ictal Semiology (Blume et al., 2001).

**Table 6:** Names of clinical signs during episodes

Elementary motor	Automatisms	Autonomic
Tonic	Dacrystic	Cardiovascular
Dystonic	Dysphasic	Gastrointestinal
Epileptic spasm	Dyspraxic	Genital
Postural	Gelastic	Hypersalivation
Versive	Gestural	Pupillary
Myoclonic	Hyperkinetic	Respiratory/apneic
Clonic	Hypokinetic	Sudomotor
Jacksonian march	Manual or pedal	Thermoregulatory
Negative myoclonic	Mimetic	Urinary incontinence
Tonic–clonic	Oroalimentary	Vasomotor
Figure-of-four: extended elbow: left/right	Vocal	Other (free text)
	Verbal	
	With preserved responsiveness	
Atonic		
Astatic	Other (free text)	
Other (free text)		

	Sensory	Experiential
Motor/behavioral arrest	Auditory	Affective/emotional
	Autonomic	Hallucinatory
Dyscognitive (free text)	Cephalic/headache	Illusory
	Epigastric	Mnemonic déjà-vu jamais-vu
Other clinical sign (free text)	Gustatory	
	Olfactory	
Not possible to determine	Painful	Other (free text)
	Somatosensory	
	Visual	
	Other (free text)	

Somatotopic modifiers describe the part of the body where the clinical sign is manifested (Blume et al., 2001). For some of the clinical signs (for example, dacrystic, gelastic) the name determines the body part too. For others this has to be selected from the list of choices: generalized (yes/no), laterality (Left/Right/Bilateral – Symmetric/Left > Right/Right > Left), body part (Eyelid/Face/Arm/Leg/Trunk/Visceral/Hemi-), and the centricity (Axial/Proximal limb/Distal limb).

Clinical and behavioral signs of ictal cognitive disturbances should be examined and recorded by testing with a standardized protocol assessing the state of consciousness (reactivity and orientation), memory, speech or language, motor and other neurologic functions of the patient (Velis et al., 2007).

*The ictal EEG pattern* is described by its name and the localization. The names selectable for ictal patterns are shown in Table 7 and defined in Appendix S5. Where indicated, frequency (Hz) and amplitude (μV) values can be specified by entering the corresponding numbers.

The localization for these patterns is scored as described above.

**Table 7:** Names of the ictal EEG patterns

Burst-suppression pattern	Low-voltage fast activity (Hz)	Sharp-and-slow-waves (Hz)
DC-shift	Obscured by artifacts	Slow wave of large amplitude (μV)
Disappearance of ongoing activity	Polysharp-waves	Spike-and-slow-waves (Hz)
Electrodecremental change	Polyspikes	Spike-and-slow-wave-runs
Fast spike activity/repetitive spikes (Hz)	Polyspike-and-slow-waves (Hz)	Classical 3/s
Irregular delta/theta activity (Hz)	Rhythmic activity (Hz)	Slow 1–2.5/s
		Fast 4–5/s
Other pattern (free text)		
No demonstrable ictal EEG change		
Not possible to determine		

*For the postictal phase*, the list of clinical signs and the list of EEG patterns are different from the onset and the propagation phases. The possible choices are shown in Table 8. The postictal EEG patterns are defined in Appendix S5. The names of the clinical signs are selected from the list according to the ILAE Commission Report: Glossary of Descriptive Terminology for Ictal Semiology (Blume et al., 2001).

**Table 8:** Names of the postictal clinical signs and EEG patterns

Postictal clinical signs		Postictal EEG patterns
Anterograde amnesia	Paresis (Todd's palsy)	Flattening
Aphasia/dysphasia	Postictal sleep	Increase in the interictal epileptiform discharges
Behavioral change	Quick recovery of consciousness	
Dysphoria		Periodic epileptiform discharges
Headache	Retrograde amnesia	
Hemianopia	Unconscious	Slowing (Hz)
Impaired cognition	Unilateral motor phenomena/myoclonia	Other (free text)
Nose wiping		
	Other unilateral motor phenomena	

In the postictal period, the clinical signs have as attribute the somatotopic modifiers. The names of the postictal EEG patterns have localization as an attribute (similarly to the onset and propagation phases).

### Physiologic patterns and patterns of uncertain significance

These items are not considered abnormal, and they are scored only if the physician finds a clinical relevance for it (for example, emphasizing that they are not abnormal/correcting the previous scoring of a junior physician, and so on). Patterns of uncertain significance contain graphoelements/EEG patterns that resemble abnormal ones, but in most of the cases they are not associated with a pathologic process (“normal variants”).

These items are described by two features: the name and the localization. The list of the names and their definitions are in Appendix S6. Localization is described as detailed above.

### Artifacts

In this SCORE element one can document the names (Appendix S7) and localization of the artifacts, and one can estimate the consequence of the artifacts on the recording: not interpretable because of the artifacts/recording of reduced diagnostic value due to artifacts/does not interfere with the interpretation of the recording.

### Polygraphic channels

In this part one can register the features related to the additional (polygraphic) sensors: electrocardiography (ECG), respiration measurements, electromyography (EMG). The possible choices are shown in Table 9. Values (numbers) are entered, where specified, in the brackets.

**Table 9:** Features and choices for the polygraphic channels

Type of sensor	Description	Significance in relation to the clinical event	Other features
ECG	Normal rhythm	Cause	QT period (value)
	Asystolia (duration: range in seconds)	Consequence	
	Bradycardia (beats/min: range)	No connected clinical episode	
	Extrasystole	Undetermined	
	Tachycardia (beats/min: range)		
	Other (free text)		

Respiration	Apnea; duration (range in seconds)	Related to EEG/clinical episode	Saturation (%)
	Hypopnea; duration (range in seconds)	Unrelated to EEG/clinical episode	
	Apnea-hypopnea index (events/h)	Not possible to determine	
	Periodic respiration		
	Tachypnea (frequency)		
	Other (free text)		

EMG	Activity unrelated to the clinical event		Localization
	Asymmetric activation of EMG (right first/left first)		Side (left, right, bilateral)
	Decreased activity related to the clinical event		Name of muscle(s) (free text
	Increased activity related to the clinical event		
	Myoclonus		
	Negative myoclonus		
	Related to EEG paroxysms		
	Rhythmic		
	Arrhythmic		
	Synchronous		
	Asynchronous		
	Periodic limb movements in sleep (PLMS)		
	Spasm		
	Tonic contraction		
	Other (free text		

EOG	Comments (free text)		
	Other sensor (free text)		

ECG, electrocardiography; EMG, electromyography; EOG, electrooculography.

### Diagnostic significance

Before generating the report, the physician has to put the scored EEG features into the clinical context. The diagnostic significance of the recording offers three choices: normal, no definite abnormality, and abnormal. If abnormal is selected, one has to specify it in more detail (Table 10).

**Table 10:** Diagnostic significance

EEG findings in keeping with			
Epilepsy	Not further specified		
	
	Status epilepticus	Focal	Convulsive
		Bilateral/generalized	Nonconvulsive
		
		Subtle	
	
	Focal	Idiopathic	Possible
	Multifocal	Symptomatic	Probable
	Generalized	Undetermined	Definite
	Undetermined		
		
	CSWS		
	
PNES			

Other nonepileptic clinical episode (free text)			
Focal CNS dysfunction			
Diffuse CNS dysfunction			
Coma			
Brain death			
EEG abnormality of uncertain clinical significance			

For “epilepsy,” further scoring of significance is available. The entries that can be selected here are not a formal classification of epilepsies, but rather highlight the additional diagnostic information the EEG can provide in the corresponding clinical context, as an element in the diagnostic workup.

In this part one can score the changes since the last (previous) EEG: no change/improved/worsened.

### Generating the report

When the scoring is done, the report is automatically generated. The physician can review, edit, or change any part of the scoring until the report is electronically signed and saved. In the report-generating window, free-text parts can be added, and a text box for “summary” can be filled in. This entry has a flexible format, where the personal wisdom of the electroencephalographer can be distilled, and the reasons for even syndrome diagnosis can be explained.

### Specific aspects of the neonatal recordings

For newborns (neonatal period = first 28 days after birth) the gestational age (GA) at birth is specified, and the GA at the time of the recording is calculated. A specific neonatal matrix is loaded instead of the “background activity” and “sleep.” This matrix contains the specific features of the neonatal ongoing activity and the characteristic transients.

The main elements are: behavioral stages (alertness), temporal organization, and spatial organization. For each entry (choice) of the behavioral stage, one can attribute temporal organization and subsequently spatial organization. These are scored further by their specific features. Temporal organization characterizes the changes in time of the ongoing EEG activity. The spatial organization codes the characteristic neonatal EEG patterns (also including transients), and their parameters—including location.

For all features scored within the temporal and spatial organization, the electroencephalographer has the possibility to label them as “considered normal for age” or “considered abnormal for age.”

The content of (selectable terms for) “behavioral stages” and “temporal organization” of the neonatal matrix is different for the conceptual age ≤30 and >30 weeks (Table 11). Definitions for the neonatal terms are presented in Appendix S8.

**Table 11:** Names for the specific, neonatal features

Behavioral stages		Temporal organization		
		
≤30 weeks	>30 weeks	≤30 weeks	>30 weeks	Spatial organization
Active	Wakefulness (eyes open)	Continuous tracing	Continuous tracing	Delta
Quiet		Discontinuous tracing	Tracé alternant	Theta
Active and quiet	Active sleep	With physiologic bursts	Discontinuous tracing	Alpha
Not possible to determine	Quiet sleep	With nonphysiologic bursts	With physiologic bursts	Beta
	Intermediate	Suppression	With nonphysiologic bursts	STOPs
	Induced state	Burst	Suppression	Delta brushes
	Not possible to determine	Continuous epileptiform activity	Burst	Premature temporal theta
		Electrocerebral inactivity	Continuous epileptiform activity	Frontal sharp transients
			Electrocerebral inactivity	Slow anterior dysrhythmia

#### Temporal organization

If “isoelectric EEG” is selected, no further scoring is available within the neonatal matrix (only “artifacts,” “polygraphic channels,” and “diagnostic significance” can be scored). If continuous tracing (tracé continu) is selected, no further specifications for the temporal organization are available in this entry. If discontinuous tracing (tracé discontunu), tracé alternant, or suppression-burst is selected, this has to be further characterized by the “duration of low-voltage interval (interburst interval)” and the “duration of electric activity (bursts).”

#### Spatial organization

“Spatial organization” is attached for each entry of “temporal organization” (except for “isoelectric EEG”). This contains: the name of the EEG pattern/graphoelement (Table 1), frequency and amplitude entries, a localization-descriptor (including also bilateral synchrony and amplitude asymmetry descriptors, as detailed above), incidence, and reactivity. The spatial organization is scored as considered normal or abnormal for age. Reactivity is scored as yes/no (the type of stimulus can be specified here in a free-text entry).

If “discontinuous tracing with nonphysiologic bursts” is selected, that means that the EEG patterns during the period of the electric activity are considered “abnormal.” In this case the list of “names” offered is not the one presented in Table 1, but the one presented in Table 3 (interictal patterns).

There is an option for a “simplified” description of the spatial organization, which contains only a global assessment of the significance and the reactivity of all graphoelements attached to that entry.

## Discussion

We constructed the SCORE software for standardized assessment, interpretation, and reporting of EEG, based on a European consensus process. The software has been tested in the clinical practice, corrected, and adjusted. Generating a report automatically feeds the scored EEG features into the database. A free version of the software is available. We plan a revision of SCORE based on the incoming suggestions, and a broader, international consensus.

In the current version of the software the database is produced locally. We plan to make an international database, where centers wishing to participate can upload their data, after appropriate processing (removing) of personal data. The legal background for data transfer has to be clarified before proceeding to this (rules and regulations unfortunately differ even within the European Union). Such an international database would constitute a valuable tool for further research projects, as search criteria can be constructed to verify hypothesis or extract relevant information from the database. In addition, the software makes it possible to compare the scored features in the report with a scored “second opinion” from another laboratory on an EEG laboratory. This offers the tools for quality control and audit.

SCORE will be helpful in bridging the gap between the classical method of visual analysis of the EEG and the advanced (computerized) analysis methods. The appropriate analysis tools can be attached to the corresponding elements of SCORE (for example quantitative EEG analysis method for “background activity”; source analysis methods for the “localization descriptor,” and so on). The electroencephalographer can enrich with these methods his armamentarium for the analysis and interpretation of EEG recordings in the clinical practice, by integrating their results in the standardized EEG report.

Integration of SCORE with the patient administration systems of the hospitals is going to save considerable time and increase the feasibility.

The terms/features used in SCORE are provided with a definition in the current version. The intention of the SCORE consortium is to provide (besides the definition) typical examples of EEG samples (screen shots) showing the various features. Therefore, in addition to the definition, an EEG sample will be accessible directly (from the feature in question) in the software—the user will be able to open this while scoring the EEG. We consider that this has remarkable potential in training neurophysiologists.

In addition to EEG, MEG data will be integrated for standardized analysis in collaboration with the European Clinical MEG Society (EMEGS).

At present the following languages are available in the software: English, Chinese, German, Dutch, Norwegian, Turkish, and Danish. Translation into eight other languages is already in progress. One can score a recording using one language and print out the report in another language.

Unfortunately most of the terms and features of the EEG report are still largely based on tradition, and systematic evaluations of their diagnostic significance are not yet available. An international EEG database would help in further, evidence-based evaluation (and ultimately selection) of the features traditionally included in the EEG report. Therefore, we plan a periodical revision of SCORE, based on these data, and on additional, incoming suggestions and comments.

## Supporting Information

**Appendix S1.** Definitions of the terms in the SCORE main flowchart.

**Appendix S2.** Definitions of terms in background activity.

**Appendix S3.** Definitions of the terms in the sleep element of SCORE.

**Appendix S4.** Definitions of terms in interictal findings.

**Appendix S5.** Definitions of terms in episodes.

**Appendix S6.** Definitions of terms in physiologic patterns.

**Appendix S7.** Definitions of terms in artifacts.

**Appendix S8.** Definitions of terms in neonatal features.
