# Supplementary material for: Standardized Computer-based Organized Reporting of EEG: SCORE
Source: Epilepsia. 2013 Mar 18;54(6):1112–24. doi: 10.1111/epi.12135 (PMC3759702; doi:10.1111/epi.12135)
Supplement: Supplementary file 1 [file epi0054-1112-SD1.pdf]

Definitions of the terms in the SCORE main flowchart

**Term**	**Definition**
Background activity	An EEG activity representing the setting in which a given normal or abnormal pattern appears and from which such pattern is distinguished.
Episodes	Clinical episodes or electrographic seizures
Modulators	External stimuli / interventions or changes in the alertness level (sleep) that modify: the background activity, or how often a graphoelement is occurring, or change other features of the graphoelement (like intra-burst frequency).<br>Examples of modulators are: hyperventilation, intermittent photic stimulation, administration of a medication during the recording.
Interictal findings	EEG patterns / transients that are distinguished form the background activity, considered abnormal, but are not recorded during ictal period (seizure) or postictal period; the presence of interictal findings does not necessarily imply that the patient has epilepsy.
Patterns of uncertain significance	EEG graphoelements or rhythms that resemble abnormal patterns but that are not necessarily associated with a pathology, and the physician does not consider them abnormal in the context of the scored recording (like normal variants and patterns).
Physiologic patterns	EEG graphoelements or rhythms that are considered normal. They only should be scored if the physician considers that they have a specific clinical significance for the recording.
Sleep and drowsiness	The features of the “ongoing” activity during sleep are scored here. If abnormal graphoelements appear, disappear or change their morphology during sleep, that is not scored here but at the entry corresponding to that graphooelement (as a modulator).
