# Supplementary material for: Standardized Computer-based Organized Reporting of EEG: SCORE
Source: Epilepsia. 2013 Mar 18;54(6):1112–24. doi: 10.1111/epi.12135 (PMC3759702; doi:10.1111/epi.12135)
Supplement: Supplementary file 2 [file epi0054-1112-SD2.pdf]

Background activity

Term	Definition
Alpha activity	EEG rhythm in the alpha range (8-13 Hz) which is considered part of the background (ongoing) activity but does not fulfil the criteria of the posterior dominant rhythm (alpha rhythm).
Amplitude asymmetry	A difference in amplitude between the homologous area on opposite sides of the head that consistently exceeds 50%.
Beta activity	EEG rhythm between 14 and 40 Hz, which is considered part of the background (ongoing) activity but does not fulfil the criteria of the posterior dominant rhythm. Most characteristically: a rhythm from 14 to 40 Hz recorded over the fronto-central regions of the head during wakefulness. Amplitude of the beta rhythm varies but is mostly below 30 µV. Other beta rhythms are most prominent in other locations or are diffuse.
Delta activity	EEG rhythm in the delta (under 4 Hz) range that does not belong to the posterior dominant rhythm (scored under other organised rhythms).
Electrocerebral inactivity	Absence of any ongoing cortical electric activities; in all leads EEG is isoelectric or only contains artefacts. Sensitivity has to be increased up to 2 µV/mm; recording time: at least 30 minutes.
Generalised suppression burst	EEG pattern consisting of bursts (activity appearing and disappearing abruptly) interrupted by periods of low amplitude (below 20 µV) and which occurs simultaneously over all head regions.
Mu rhythm	EEG rhythm at 7-11 Hz composed of arch-shaped waves occurring over the central or centro-parietal regions of the scalp during wakefulness. Amplitudes varies but is mostly below 50 µV. Blocked or attenuated most clearly by contralateral movement, thought of movement, readiness to move or tactile stimulation.
Other organised rhythms of the background activity	EEG activity that consisting of waves of approximately constant period, which is considered as part of the background (ongoing) activity, but does not fulfil the criteria of the posterior dominant rhythm.
Posterior dominant rhythm	Rhythmic activity occurring during wakefulness over the posterior regions of the head, generally with maximum amplitudes over the occipital areas. Amplitude varies. Best seen with eyes closed and during physical relaxation and relative mental inactivity. Blocked or attenuated by attention, especially visual, and mental effort.

	In adults this is the alpha rhythm, and the frequency is 8 – 13 Hz. However the frequency can be higher or lower than this range (often a supra or sub-harmonic of alpha frequency) and is called alpha variant rhythm (fast and slow alpha variant rhythm).<br>In children, the normal range of the frequency of the posterior dominant rhythm is age-dependant.
Reactivity	Susceptibility of individual rhythms or the EEG as a whole to change following sensory stimulation or other physiologic actions. (The type of stimulus has to be specified in free-text).
Reactivity to eye opening / closure	Change (disappearance or measurable decrease in amplitude) of a posterior dominant rhythm following eye-opening. Eye closure has the opposite effect.
Suppression (of background activity)	Periods showing activity under 10 μV (referential montage) and interrupting the background (ongoing) activity.
Theta activity	EEG rhythm in the theta (4-8 Hz) range that does not belong to the posterior dominant rhythm (scored under other organised rhythms).
