# Supplementary material for: Standardized Computer-based Organized Reporting of EEG: SCORE
Source: Epilepsia. 2013 Mar 18;54(6):1112–24. doi: 10.1111/epi.12135 (PMC3759702; doi:10.1111/epi.12135)
Supplement: Supplementary file 3 [file epi0054-1112-SD3.pdf]

Definitions of the terms in the sleep element of SCORE

**Term**	**Definition**
Abnormal asymmetry or absence of sleep graphoelements	Absence or consistently marked amplitude asymmetry (>50%) of a normal sleep graphoelement.
Hypnagogic /hypnopompic hypersynchrony in children	Bursts of bilateral, synchronous delta or theta activity of large amplitude, occasionally with superimposed faster components, occurring during falling asleep or during awakening, in children.
K complex	A burst of somewhat variable appearance, consisting most commonly of a high voltage negative slow wave followed by a smaller positive slow wave frequently associated with a sleep spindle. Duration > 0.5 s. Amplitude is generally maximal in the frontal vertex. K complexes occur during non-REM sleep, apparently spontaneously, or in response to sudden sensory / auditory stimuli, and are not specific for any individual sensory modality.
Non-reactive sleep activity.	EEG activity consisting of normal sleep graphoelemts, but which cannot be interrupted by external stimuli/ the patient cannot be waken.
Normal sleep pattern	Recording containing sleep-patterns that are considered normal for the attained sleep stages and for the age.
Positive occipital sharp transient of sleep	Sharp transient maximal over the occipital regions, positive relative to other areas, apparently occurring spontaneously during sleep. May be single or repetitive. Amplitude varies but is generally bellow 50 µV.
Sleep architecture	Only to be scored if whole-night sleep is part of the recording. It is a global descriptor of the structure and pattern of sleep: estimation of the amount of time spent in REM and NREM sleep, sleep duration, NREM-REM cycle.
Sleep onset rapid eye movement sleep (SOREM)	Occurrence of REM (rapid eye movement) sleep within 15 minutes after sleep onset.
Sleep spindles	Burst at 11-15 Hz but mostly at 12-14 Hz generally diffuse but of higher voltage over the central regions of the head, occurring during sleep. Amplitude varies but is mostly below 50 µV in the adult.
Vertex sharp transient	Sharp potential, maximal at the vertex, negative relative to other areas, apparently occurring spontaneously during sleep or in response to a

	sensory stimulus during sleep or wakefulness. May be single or repetitive. Amplitude varies but rarely exceeds 250 μV. Abbreviation: V wave. Synonym: vertex sharp wave.
