# Supplementary material for: Standardized Computer-based Organized Reporting of EEG: SCORE
Source: Epilepsia. 2013 Mar 18;54(6):1112–24. doi: 10.1111/epi.12135 (PMC3759702; doi:10.1111/epi.12135)
Supplement: Supplementary file 4 [file epi0054-1112-SD4.pdf]

INTERICTAL FINDINGS

Epileptiform Discharges

Transients distinguishable from background activity, with characteristic spiky morphology, typically, but neither exclusively, nor invariably found in interictal EEGs of people with epilepsy

Term	Definition
Polyspike-and-slow-wave complex	Two or more consecutive spikes associated with one or more slow waves.
Polyspikes	Two or more consecutive spikes.
Runs of rapid spikes	Bursts of spike discharges at a rate from 10 to 25/sec (in most cases somewhat irregular). The bursts last more than 2 seconds (usually 2 to 10 seconds) and it is typically seen in sleep.<br>*Synonyms:* rhythmic spikes, generalized paroxysmal fast activity, fast paroxysmal rhythms, grand mal discharge, fast beta activity
Sharp wave	A transient clearly distinguished from background activity, with pointed peak at a conventional paper speed or time scale, and duration of 70-200 ms, i.e. over ¼-1/5 s approximately. Main component is generally negative relative to other areas. Amplitude varies.<br>Comments:<br>1. Term should be restricted to epileptiform discharges and does not apply to (a) distinctive physiologic events such as vertex sharp transients, lambda waves, and positive occipital sharp transients of sleep, (b) sharp transients poorly distinguished from background activity and sharp-appearing individual waves of EEG rhythms.

	2. Sharp waves should be differentiated from spikes, i.e. transients having similar characteristics but shorter duration. However, it should be kept in mind that this distinction is largely arbitrary and primarily serves descriptive purposes.
Sharp-and-slow-wave complex	A sequence of a sharp wave and a slow wave.
Slow sharp wave	A transient that bears all the characteristics of a sharp-wave, but exceeds 200 ms. Synonym: blunted sharp wave.
Spike	A transient, clearly distinguished from background activity, with pointed peak at a conventional paper speed or time scale and duration from 20 to under 70 ms, i.e. 1/50-1/15 s approximately. Main component is generally negative relative to other areas. Amplitude varies.<br>Comments:<br>1. Term should be restricted to epileptiform discharges. EEG spikes should be differentiated from sharp waves, i.e. transients having similar characteristics but longer duration. However, it should be kept in mind that this distinction is largely arbitrary and primarily serves descriptive purposes.<br>2. A slow negative component may trail the spike discharge and often attain the same amplitude as the negative main component of the spike. This trailing component of a single spike should not be regarded as evidence of a spike-and-slow-wave complex.
Spike-and-slow-wave	A pattern consisting of a spike followed by a slow wave
Spike-and-slow-wave runs:	
- Classical 3/s ~	Characteristic paroxysm consisting of a regular sequence of spike-and-slow-wave complexes which: (1) repeat at 3-3.5 c/s (measured during the first few seconds of the paroxysm), (2) are bilateral in their onset

	and termination, generalized, and usually of the maximum amplitude over the frontal areas, (3) are approximately synchronous and symmetrical on the two sides of the head throughout the paroxysm.
- Slow 1-2.5/s ~	Spike-and-slow-wave complexes which consist of a rather slow spike (according to definition, a sharp wave, lasting 70 msec or longer and a slow wave), repeat at 1-2.5 Hz frequency, and are bilateral synchronous (generalized) with a frontal midline maximum.
- Fast 4-5/s ~	Spike-and-slow-wave complexes which repeat at 4 or 4-5/s, are bilateral, usually of the maximum amplitude over the frontal areas, are approximately synchronous and symmetrical on the two sides of the head throughout the paroxysm.
- Atypical ~	Paroxysms consisting of an irregular sequence of spike-and-slow-wave complexes, that occur bilaterally but do not meet the criteria of the above described subtypes.

Abnormal slow activity

Activity of frequency lower than alpha, that clearly exceeds the amount considered physiologically normal for the patient's age and state of alertness.

**Term**	**Definition**
Delta activity	Rhythm with frequency of under 4 Hz; clearly exceeds the amount considered physiologically normal for the patient's age and state of alertness.
Delta and theta activity	Quasi-rhythmic activity of frequencies in the delta and theta range.
Intermittent	A slow activity that occurs intermittently, in short bursts, and it is not caused by drowsiness. It is usually

rhythmic slow activity	rhythmic and often in the delta frequency range, thus accounting for the term: IRDA (intermittent rhythmic delta activity). When in the delta frequency range, it is often composed of runs of sinusoidal or saw-toothed waves with more rapid ascending then descending phases, with mean frequencies close to 2.5 Hz. It is usually attenuated by alertness and eye opening, and accentuated with eye closure, hyperventilation and drowsiness.
- FIRDA	Frontal intermittent rhythmic delta activity<br>Fairly regular or approximately sinusoidal waves, mostly occurring in bursts at 1.5-2.5 Hz over the frontal areas of one or both sides of the head. Comment: most commonly associated with unspecified encephalopathy, in adults.
- OIRDA	Occipital intermittent rhythmic delta activity<br>Fairly regular or approximately sinusoidal waves, mostly occurring in bursts at 2-3 Hz over the occipital or posterior head regions of one or both sides of the head. Frequently blocked or attenuated by opening the eyes. Comment: most commonly associated with unspecified encephalopathy, in children
-TIRDA	Temporal intermittent rhythmic delta activity<br>Fairly regular or approximately sinusoidal waves, mostly occurring in bursts at over the temporal areas of one side of the head. Comment: most commonly associated with temporal lobe epilepsy.
Polymorphic delta activity	EEG activity consisting of waves in the delta range (over 250 ms duration for each wave) but of different morphology.
Theta activity	Rhythm with frequency of 4 to under 8 Hz; clearly exceeds the amount considered physiologically normal for the patient’s age and state of alertness.

Special patterns

Term	Definition
Bi-PLEDS	PLEDs that occur independently over both hemispheres. See also: PLEDs.
Bursts suppression pattern	Pattern characterized by burst of theta and/or delta waves, at times intermixed with faster waves, and intervening periods of low amplitude (below 20µV). Comment: EEG pattern that indicates either severe brain dysfunction or is typical for some anesthetic drugs at certain level of anesthesia.
Hypsarrhythmia	Pattern consisting of diffuse chaotic high voltage (>300 µV) irregular slow waves interspersed with multiregional spikes and sharp waves over both hemispheres.
PLEDs	Periodic lateralized epileptiform discharges<br>Epileptiform discharges such as spikes or sharp waves, often with multiple phases and complex morphology, which repeat in a periodical or semiperiodical fashion. They have either a regional or a lateralized distribution. The main component is negative. Comment: are usually not associated with epileptic seizure disorders characterized by chronically recurrent seizures. Thy represent the EEG feature of a severe ongoing central nervus system disease with certain paroxysmal or even overt epileptogenic properties.
Periodic complex discharges – other than PLEDs	Epileptiform discharges, often with multiple phases and complex morphology, which repeat in a periodical or semiperiodical fashion, but do not fulfill the criteria for PLEDs. They are always of large amplitude, mostly in range of 100 to 500 µV. These may be simple sharp waves, but in duration that usually exceeds 150 msec. Other periodic discharges are compounded and polymorphic. Periodic discharges may be multiregional, widely scattered, or generalized synchronous. Comment: are usually not associated with epileptic seizure disorders characterized by chronically recurrent seizures. Thy represent

	the EEG feature of a severe ongoing central nervus system disease with certain paroxysmal or even overt epileptogenic properties.
SIRPIDS	Stimulus-induced Rhythmic, periodic, or ictal discharges<br>Periodic, rhythmic, or ictal-appearing EEG discharges that were consistently induced by alerting stimuli.
Triphasic waves	High amplitude (over 70 µV) positive sharp transients, which are preceded and followed by relatively low amplitude negative waves. The first negative wave generally has a lower amplitude than the negative afterwave. The distribution is generalized, and frequently the largest deflections in a bipolar fronto-occipital derivation occur at the frontal electrodes. Triphasic waves tend to have a repetition rate of ca. 1-2 Hz.

Neonatal interictal patterns

Alpha bursts	Non-ictal epochs of alpha activity
Brief interictal rhythmic discharges	Short non-ictal periods of rhythmic activity
Positive Rolandic sharp waves (PRSW)	Positive sharp waves in premature infants with intraventricular hemorrhages, chiefly over Rolandic area
Positive temporal spikes (PTS)	Transients with morphology and polarity similar to those of positive PRSWs but localized to the temporal areas. They occur in neonates with intracranial hemorrhages or a history of perinatal asphyxia.

Time-related features

Term	Definition
Arhythmic trains/ bursts	Applies to the intra-burst pattern: a non-ictal graphoelement that repeats itself without returning to the background activity between them. The graphoelements within this repetition occur at inconstant period.
Discharge pattern	Describes the organization of the EEG signal within the discharge (distinguishes between singe discharges and repetitive discharges).
Mode of appearance	Describes how the non-ictal EEG pattern / graphoelement is distributed throughout the recording
Periodic	Non-ictal EEG pattern / graphoelement occurring at an approximately regular rate / interval (generally of 1 to several seconds).
Random	Occurrence of the the non-ictal EEG pattern / graphoelement without any rhytmicity / periodicity
Rhytmic trains/ bursts	Applies to the intra-burst pattern: a non-ictal graphoelement that repeats itself without returning to the background activity between them. The graphoelements within this repetition occur at approximately constant period.
Single discharge	Applies to the intra-burst pattern: a graphoelement that is not repetitive; before and after the graphoelement one can distinguish the background activity.
Variable	Occurrence of non-ictal EEG pattern / graphoelements, that is sometimes rhythmic or periodic, other times random, throughout the recording
