# Supplementary material for: Standardized Computer-based Organized Reporting of EEG: SCORE
Source: Epilepsia. 2013 Mar 18;54(6):1112–24. doi: 10.1111/epi.12135 (PMC3759702; doi:10.1111/epi.12135)
Supplement: Supplementary file 5 [file epi0054-1112-SD5.pdf]

EPISODES

NAMES OF EPISODES

Names	Definitions
**GENERALIZED EPILEPTIC SEIZURES**	
**Absence***:* - *Atypical*	Absence seizures may have a) change in tone that are more pronounced than in typical absences; b) onset and /or cessation that is not abrupt. Ictal EEG: it is more heterogeneous; may include irregular spike-and-slow wave complexes, fast activity or other paroxysmal activity. Abnormalities are bilateral but often irregular and asymmetrical. Interictal EEG: background is usually abnormal; paroxysmal activity (such as spikes and spike-and-slow wave complexes) frequently irregular and asymmetrical.
**Absence***:* - *Typical*	Clinical manifestations can be: a) impairment of consciousness only; b) with mild clonic components; c) with atonic components; d) with tonic components; e) with automatisms; f) with autonomic components (b through f may be used alone or in combination). Ictal EEG: usually regular and symmetrical 3 Hz but may be 2-4 Hz spike-and-slow complexes. Abnormalities are bilateral. Interictal EEG: background activity usually normal although paroxysmal activity (such as spikes or spike-and-slow-wave complexes) may occur. This activity is usually regular and symmetrical.
**Atonic**	Seizure characterized by sudden loss or diminution of muscle tone without apparent preceding myoclonic or tonic event lasting $\geq$ 1 to 2 s, involving head, trunk, jaw, or limb musculature.
**Clonic**	Seizure characterized by myoclonic muscle jerks that are regularly repetitive, involve the same muscle groups, at a frequency of ~2–3 c/s, and are prolonged. Synonym: rhythmic myoclonus. Ictal EEG: fast activity (10 Hz or more) and slow waves; or: spike-and wave pattern.

**Eyelid myoclonia** - *with (or without) absence*	Episodes characterized by occurrence of eyelid myoclonia, i.e. marked jerking of the eyelids immediately after eye closure with or without brief absences, usually lasting less than 6 sec. Ictal EEG: high-amplitude generalized polyspikes or polyspikewave complexes, often followed by brief discharges (3–6 s or less) of rhythmic spike- or polyspike-wave complexes at 3 or more per second. Typically, these abnormalities are triggered by active eye-closure (not by simple eye blinks), occurring immediately (usually about 0.5s) on closing the eyes, in a well-lit recording room; darkness abolishes totally or partially this response, whereas hyperventilation can enhance generalized paroxysmal activity and eventually give rise to eyelids myoclonia and absences. Interictal EEG: generalized photoparoxysmal EEG response
**Myoclonic**	Episode characterized by sudden, brief (<100 ms) involuntary single or multiple contraction(s) of muscles(s) or muscle groups of variable topography (axial, proximal limb, distal) (Blume et al., 2001). Generalized myoclonic episodes may be associated with bilateral, symmetrical spike/polyspike wave complexes or sharp and slow waves
**Myoclonic absence**	Absence seizure characterized by abrupt onset associated with bilateral rhythmic myoclonic jerks of severe intensity. The loss of consciousness during the absence may be complete or partial. The seizure mainly involves muscles of the shoulders, arms, and legs; facial muscles are less involved. When facial myoclonias occur, they are more evident around the chin and mouth, whereas eyelid twitching is typically absent or rare. The movements may be sustained and progressive, being associated with tonic contraction, which is maximal in shoulder and deltoid muscles. The jerks and tonic contractions may be symmetrical or predominant on one side, causing turning of the head and body. Autonomic manifestations such as arrest of respiratory movement and urinary incontinence may also be present. Each episode of myoclonic absences may last from 10 to 60 seconds. Ictal EEG in epilepsy with myoclonic absences shows a pattern of bilateral, synchronous, and

	symmetrical discharge of spike-waves at 3 Hz, similar to that of childhood absences. The discharges may end with delta waves in frontal areas, which may be asymmetrical. The spike-wave discharges may be interspersed with polyspike and wave activity. Polygraphic recording of myoclonic absences discloses the appearance of bilateral myoclonias, at the same frequency as the spikes and waves, which begin 1 or 2 seconds after the onset of the EEG paroxysmal discharges and are followed by a tonic, sometimes asymmetrical, contraction, maximal in the deltoid and shoulder muscles. Interictal EEG findings include normal background activity in all cases with superimposed generalized spikes and waves or, more rarely, focal or multifocal spikes and waves. Photosensitivity is uncommon. The sleep EEG shows a normal organization and symmetrical physiological patterns. During sleep the evolution of the spikes and waves is similar, on the whole, to that observed in childhood absence epilepsy
**Myoclonic atonic**	Seizures in which the atonia is preceded by a myoclonic jerk (i.e., a sudden and brief – less than 100 msec – involuntary contraction of muscle groups of variable topography. Falling seizures are better termed *astatic,* this term implying “loss of erect posture that results from atonic, myoclonic or tonic mechanisms”; drop attacks would be a synonym. Ictal EEG: polyspikes and waves or flattening or low-voltage fast activity
**Myoclonic tonic**	Seizure characterized by a myoclonic phenomenon followed by a tonic phase.
**Tonic**	Seizure characterized by sustained increase in muscle contraction lasting a few seconds to minutes. Ictal EEG: low-voltage fast activity or a fast rhythm of 9-10 c/sec or more decreasing in frequency and increasing in amplitude
**Tonic–clonic (in any combination)**:	A sequence consisting of a tonic followed by a clonic phase. Variants such as clonic–tonic–clonic may be seen. *Generalized tonic-clonic seizure*: Bilateral symmetric tonic contraction and then bilateral clonic contractions of somatic muscles, usually associated with autonomic

	phenomena.
**FOCAL EPILEPTIC SEIZURES**	
Localization	It refers to the initial activation of a system of neurons limited to part of one cerebral hemisphere as indicated by the first clinical and EEG changes<br>*Frontal / Temporal / Rolandic / Parietal / Occipital:* it refers to seizures whose ictal signs and symptoms suggest the predominant involvement of a circumscribed brain region defined according to brain lobes
Evolving to bilateral convulsive seizure:	Involves tonic, clonic, or tonic and clonic components. A focal seizure propagating to a wide cortical network, both hemispheres. This expression replaces the term ''secondarily generalized seizure''.
**OTHER TYPES OF EPILEPTIC SEIZURES**	
**Electrographic seizure**	Referred usually to non convulsive status. Ictal EEG: rhythmic discharge or spike and wave pattern with definite evolution in frequency, location, or morphology lasting at least 10 s; evolution in amplitude alone did not qualify
**Epileptic spasm**	A sudden flexion, extension, or mixed extension–flexion of predominantly proximal and truncal muscles that is usually more sustained than a myoclonic movement but not so sustained as a tonic seizure (i.e., ~1 s). Limited forms may occur: grimacing, head nodding. Epileptic spasms frequently occur in clusters. Ictal EEG demonstrates a generalized low-amplitude fast activity or high amplitudes slow waves for seconds.
**Subtle seizure**	Seizure type frequent in neonates, sometimes referred to as motor automatisms; they may include random and roving eye movements, sucking, chewing motions, tongue protrusion, rowing or swimming or boxing movements of the arms, pedaling and bycicling movements of the lower limbs; apneic seizures are relatively common. Although some subtle seizures are associated with rhythmic ictal EEG discharges **,** and are clearly epileptic, ictal EEG often does not show typical epileptic activity
**Tonic spasm**	Sudden axial contraction as in epileptic spasms, followed by a tonic

	phase of up to 10 seconds.
**OTHER EPISODES**	
**Cataplexy**	A sudden decrement in muscle tone and loss of deep tendon reflexes, leading to muscle weakness, paralysis, or postural collapse. Cataplexy usually is precipitated by an outburst of emotional expression—notably laughter, anger, or startle. It is one of the tetrad of symptoms of narcolepsy. During cataplexy, respiration and voluntary eye movements are not compromised. Consciousness is preserved.
**PNES (Psychogenic non-epileptic seizure)**	Paroxysmal events that mimic (or are confused with) epileptic seizures, but which do not result from epileptic activity; they lack the EEG ictal findings during the ictus
**Syncope**	Episode with loss of consciousness and muscle tone that is abrupt in onset, of short duration and followed by rapid recovery; it occurs in response to transient impairment of cerebral perfusion. Typical prodromal symptoms often herald onset of syncope and postictal symptoms are minimal. Syncopal convulsions resulting from cerebral anoxia are common but are not a form of epilepsy, nor are there any accompanying EEG ictal discharges.
**SLEEP RELATED EVENTS**	
**Benign sleep myoclonus:**	A distinctive disorder of sleep characterized by a) neonatal onset, b) rhythmic myoclonic jerks only during sleep and c) abrupt and consistent cessation with arousal, d) absence of concomitant electrographic changes suggestive of seizures, and e) good outcome.
**Confusional awakening**	Episodes of non epileptic nature included in NREM parasomnias, characterized by sudden arousal and complex behavior but without full alertness, usually lasting a few minutes and occurring almost in all children at least occasionally. Amnesia of the episode is the rule.
**PLMS (Periodic Limb Movement in Sleep)**	Episodes characterized by brief (0.5- to 5.0-second) lower-extremity movements during sleep, which typically occur at 20- to 40-second intervals, most commonly during the first 3 hours of sleep. The affected individual is usually not aware of the movements or of the transient partial arousals.

**RBD (REM Sleep Behavioral Disorder)**	Episodes characterized by: a) presence of REM sleep without atonia (RSWA) on polysomnography (PSG); b) presence of at least 1 of the following conditions - (1) Sleep-related behaviors, by history, that have been injurious, potentially injurious, or disruptive (example: dream enactment behavior); (2) abnormal REM sleep behavior documented during PSG monitoring; (3) absence of epileptiform activity on electroencephalogram (EEG) during REM sleep (unless RBD can be clearly distinguished from any concurrent REM sleep-related seizure disorder); (4) sleep disorder not better explained by another sleep disorder, a medical or neurologic disorder, a mental disorder, medication use, or a substance use disorder.
**Sleep-walking**	Episodes characterized by ambulation during sleep; the patient is difficult to arouse during an episode, and is usually amnesic following the episode. Episodes usually occur in the first third of the night during slow wave sleep. Polysomnographic recordings demonstrate 2 abnormalities during the first sleep cycle: frequent, brief, nonbehavioral EEG-defined arousals prior to the somnambulistic episode and abnormally low δ (0.75-2.0 Hz) EEG power on spectral analysis, correlating with high-voltage "hypersynchronic δ" waves lasting 10 to 15 s occurring just prior to the movement. This is followed by stage I NREM sleep, and there is no evidence of complete awakening.
**PAEDIATRIC EVENTS**	
**Hyperekplexia**	Disorder characterized by exaggerated startle response and hypertonicity that may occur during the first year of life and in severe cases during the neonatal period. Children usually present with marked irritability and recurrent startles in response to handling and sounds. Severely affected infants can have severe jerks and stiffening, sometimes with breath-holding spells.
**Jactatio capitis nocturna**	Relatively common in normal children at the time of going to bed, especially during the first year of life, the rhythmic head movements persist during sleep. Usually, these phenomena disappear before 3 years

	of age.
**Paroxysmal motor event**	Paroxysmal phenomena during neonatal or childhood periods characterized by recurrent motor or behavioural signs or symptoms that must be distinguishes from epileptic disorders.
**Pavor nocturnus**	Nocturnal episodes characterized by age of onset of less than five years (mean age 18 months, with peak prevalence at five to seven years), appearance of signs of panic two hours after falling asleep with crying, screams, a fearful expression, inability to recognize other people including parents (for a duration of 5-15 minutes), amnesia upon awakening. Pavor nocturnus occurs in patients almost every night for months or years (but the frequency is highly variable and may be as low as once a month) and is likely to disappear spontaneously at the age of six to eight years.
**Stereotypical behaviour:**	Repetitive motor behavior in children, typically rhythmic and persistent; usually not paroxysmal and rarely suggest epilepsy. They include head-banging, head-rolling, jactatio capitis nocturna, body rocking, buccal or lingual movements, hand flapping and related mannerisms, repetitive hand-waving (to self-induce photosensitive seizures).

ICTAL AND POSTICTAL EEG PATTERNS

**Term**	**Definition**
Burst-suppression pattern	Pattern characterized by burst of theta and/or delta waves, at times intermixed with faster waves, and intervening periods of low amplitude (below 20μV).
DC shift	Shift of negative polarity of the direct current recordings, during seizures.
Disappearance of ongoing activity	Disappearance of the EEG activity that preceded the ictal event, but still some remnants of background activity (thus not enough to name it electrodecremental change)
Electrodecremental	Sudden desynchronization of electrical activity.

change	
Fast spike activity	A burst consisting of a sequence of spikes. Duration > 1 s. Frequency at least in the alpha range.
Flattening (postictal)	Postictal, transitory suppression of EEG activity.
Increase in the interictal epileptiform discharges (postictal)	Postictal phenomenon: transitory increase in the incidence of the inter-ictal epileptiform discharges. However, the localisation and the morphology is unchanged.
Irregular delta/ theta activity	EEG activity consisting of repetitive waves of inconsistent wave-duration but in the delta and/or theta range (>125 ms).
Low-voltage fast activity	Refers to the fast, and often recruiting activity which can be recorded at the onset of an ictal discharge, particularly in invasive EEG recording of a seizure.
Obscured by artefacts	The interpretation of the ictal EEG is not possible due to artefacts
No demonstrable ictal EEG change	Lack of change in the EEG during the clinical event.
Periodic epiletiform discharges	Epileptiform dischages (for example spikes, sharp-waves) that repeat at approximately constant period; there is a return to the background activity (not necessarily the normal background) between the successive discharges.
Polysharp-waves	A sequence of two or more sharp-waves.
Polyspikes	A sequence of two or more spikes. Duration < 1 s.
Polyspike-and-slow-wave complexes	A sequence of two or more spikes associated with one or more slow waves.
Rhythmic activity	EEG activity consisting of a sequence of waves of approximately constant period.
Sharp-and-slow-wave complexes	A sequence of a sharp wave and a slow wave.
Slowing (postictal)	A transitory postictal phenomenon: waves of longer duration (and usually higher amplitude) than of the background activity.
Spike-and-slow-wave complexes	A pattern consisting of a spike followed by a slow wave.

NAMES OF ICTAL CLINICAL FINDINGS

**Names**	**Definitions**
**ELEMENTARY MOTOR**	
Tonic	A sustained increase in muscle contraction lasting a few seconds to minutes.
Dystonic	Sustained contractions of both agonist and antagonist muscles producing athetoid or twisting movements, which, when prolonged, may produce abnormal postures
Epileptic spasm	A sudden flexion, extension, or mixed extension–flexion of predominantly proximal and truncal muscles that is usually more sustained than a myoclonic movement but not so sustained as a tonic seizure (i.e., ~1 s). Limited forms may occur: grimacing, head nodding. Frequent occurrence in clusters.
Postural	Adoption of a posture that may be bilaterally symmetric or asymmetric (as in a "fencing posture")
Versive	A sustained, forced conjugate ocular, cephalic, and/or truncal rotation or lateral deviation from the midline
Myoclonic	Characterized by myoclonus.<br>MYOCLONUS : **s**udden, brief (<100 ms) involuntary single or multiple contraction(s) of muscles(s) or muscle groups of variable<br>topography (axial, proximal limb, distal)
Clonic	Myoclonus that is regularly repetitive, involves the same muscle groups, at a frequency of ~2–3 c/s, and is prolonged. Synonym: rhythmic myoclonus
Jacksonian march	Term indicating spread of clonic movements through contiguous body parts unilaterally
Negative myoclonic	Characterized by negative myoclonus.<br>NEGATIVE MYOCLONUS: interruption of tonic muscular activity for <500 ms without evidence of preceding myoclonia.

Tonic-clonic	A sequence consisting of a tonic followed by a clonic phase. Variants such as clonic–tonic–clonic may be seen.
Generalized tonic-clonic seizure (Formerly "Grand Mal" Seizure)	Bilateral symmetric tonic contraction and then bilateral clonic contractions of somatic muscles, usually associated with autonomic phenomena.
- Figure of four: extended elbow: left/right	Asymmetry of limb posture during the tonic phase of a GTC: one arm is rigidly extended at the elbow (often with the fist clenched tightly and flexed at the wrist), whereas the opposite arm is flexed at the elbow.
Atonic	Sudden loss or diminution of muscle tone without apparent preceding myoclonic or tonic event lasting $\geq$ 1 to 2 s, involving head, trunk, jaw, or limb musculature.
Astatic	Loss of erect posture that results from an atonic, myoclonic, or tonic mechanism. Synonym: drop attack.
**AUTOMATISMS**	
Dacrystic	Bursts of crying.
Dysphasic	Impaired communication involving language without dysfunction of relevant primary motor or sensory pathways, manifested as impaired comprehension, anomia, paraphasic errors, or a combination of these.
Dyspraxic	Inability to perform learned movements spontaneously or on command or imitation despite intact relevant motor and sensory systems and adequate comprehension and cooperation
Gelastic	Bursts of laughter or giggling, usually without an appropriate affective tone.
Gestural	Semipurposive, asynchronous hand movements. Often unilateral.
Hyperkinetic	1. Involves predominantly proximal limb or axial muscles producing irregular sequential ballistic movements, such as pedaling, pelvic thrusting, thrashing, rocking movements.<br>2. Increase in rate of ongoing movements or inappropriately rapid performance of a movement.
Hypokinetic	A decrease in amplitude and/or rate or arrest of ongoing motor

	activity.
Manual or pedal	1. Indicates principally distal components, bilateral or unilateral.<br><br>2. Fumbling, tapping, manipulating movements.
Mimetic	Facial expression suggesting an emotional state, often fear.
Oroalimentary	Lip smacking, lip pursing, chewing, licking, tooth grinding, or swallowing.
Vocal	Single or repetitive utterances consisting of sounds such as grunts or shrieks.
Verbal	Single or repetitive utterances consisting of words, phrases, or brief sentences.
With preserved responsiveness	With preserved ability to carry out simple commands or willed movement.
**AUTONOMIC**	
Cardiovascular	Modifications of heart rate (tachycardia, bradycardia), cardiac arrhythmias (such as sinus arrhythmia, sinus arrest, supraventricular tachycardia, atrial premature depolarizations, ventricular premature depolarizations, atrio-ventricular block, bundle branch block, atrioventricular nodal escape rhythm, asystole)
Gastrointestinal	Nausea, eructation, vomiting, retching, abdominal sensations, abdominal pain, flatulence, spitting, diarrhoea
Genital	Sexual auras (erotic thoughts and feelings, sexual arousal and orgasm).<br>Genital auras (unpleasant, sometimes painful, frightening or emotionally neutral somatosensory sensations in the genitals that can be accompanied by ictal orgasm).<br>Sexual automatisms (hypermotor movements consisting of writhing, thrusting, rhythmic movements of the pelvis, arms and legs, sometimes associated with picking and rhythmic manipulation of the groin or genitalia, exhibitionism and masturbation).

Hypersalivation	Increase in production of saliva leading to uncontrollable drooling
Pupillary	Mydriasis, miosis (either bilateral or unilateral)
Respiratory/apnoeic	subjective shortness of breath, hyperventilation, stridor, coughing, choking, apnea, oxygen desaturation, neurogenic pulmonary edema
Sudomotor	Sweating and piloerection (may be accompanied by feelings of warmth, cold and pain)
Thermoregulatory	Hyperthermia, fever
Urinary incontinence	urinary urge (intense urinary urge at the beginning of seizures), urinary incontinence, ictal urination (rare symptom of partial seizures without loss of consciousness)
Vasomotor	Flushing or pallor (may be accompanied by feelings of warmth, cold and pain).
**MOTOR/BEHAVIOURAL ARREST**	Interruption of ongoing motor activity or of ongoing behaviours with fixed gaze, without movement of the head or trunk (oro-alimentary and hand automatisms may continue)
**DYSCOGNITIVE**	The term describes events in which (1) disturbance of cognition is the predominant or most apparent feature, and (2a) two or more of the following components are involved, or (2b) involvement of such components remains undetermined. Otherwise, use the more specific term (e.g., "mnemonic experiential seizure" or "hallucinatory experiential seizure").<br>Components of cognition:<br>• perception: symbolic conception of sensory information<br>• attention: appropriate selection of a principal perception or task<br>• emotion: appropriate affective significance of a perception<br>• memory: ability to store and retrieve percepts or concepts<br>• executive function: anticipation, selection, monitoring of consequences, and initiation of motor activity including praxis, speech
**SENSORY**	
Auditory	Buzzing, drumming sounds or single tones.
Autonomic	A sensation consistent with involvement of the autonomic nervous system, including cardiovascular, gastrointestinal,

	sudomotor, vasomotor, and thermoregulatory functions. (Thus "autonomic aura"; cf. "autonomic events" 3.0).
Headache	Headache occurring in close temporal proximity to the seizure or as the sole seizure manifestation
Epigastric	Abdominal discomfort including nausea, emptiness, tightness, churning, butterflies, malaise, pain, and hunger; sensation may rise to chest or throat. Some phenomena may reflect ictal autonomic dysfunction.
Gustatory	Taste sensations including acidic, bitter, salty, sweet, or metallic.
Painful	Peripheral (lateralized/bilateral), cephalic, abdominal
Somatosensory	Tingling, numbness, electric-shock sensation, sense of movement or desire to move.
Visual	Flashing or flickering lights, spots, simple patterns, scotomata, or amaurosis.
**EXPERIENTIAL**	
Affective/emotional	Components include fear, depression, joy, and (rarely) anger.
Hallucinatory	Composite perceptions without corresponding external stimuli involving visual, auditory, somatosensory, olfactory, and/or gustatory phenomena. Example: "hearing" and "seeing" people talking.
Illusory	An alteration of actual percepts involving the visual, auditory, somatosensory, olfactory, or gustatory systems.
Mnemonic:	Components that reflect ictal dysmnesia such as feelings of familiarity (deja`-vu) and unfamiliarity (jamais-vu).

NAMES OF POSTICTAL CLINICAL FINDINGS

Names	Definitions
Anterograde amnesia	Impaired ability to remember new material
Aphasia/dysphasia	Impaired communication involving language without dysfunction

	of relevant primary motor or sensory pathways, manifested as impaired comprehension, anomia, paraphasic errors, or a combination of these.
Behavioural changes	Occurring immediately after a seizure. They include psychosis, hypomania, obsessive-compulsive behaviour.
Dysphoria	Depression, irritability, euphoric mood, fear, anxiety.
Headache	Headache with features of tension-type or migraine headache that develops within 3 h following the seizure and resolves within 72 h after the seizure.
Hemianopia	Postictal visual loss in a hemi-field. See also: Todd’s palsy.
Impaired cognition	Decreased cognitive performance involving one or more of perception, attention, emotion, memory, execution, praxis, speech
Nosewiping:<br>left/right side of the nosewiping hand	Nosewiping usually within 60 seconds of seizure offset, usually with the hand ipsilateral to the seizure onset.
Paresis (Todd’s palsy)	Any unilateral postictal dysfunction relating to motor, language, sensory, and/or integrative functions.
Postictal sleep	Invincible need to sleep after a seizure.
Quick recovery of consciousness	Quick recovery of awareness and responsiveness.
Retrograde amnesia	Impaired ability to recall previously remembered material.
Unconscious	Unawareness and unresponsiveness
Unilateral motor phenomena	Unilateral motor phenomena, other then specified above, occurring in the postictal phase.
