# Supplementary material for: Standardized Computer-based Organized Reporting of EEG: SCORE
Source: Epilepsia. 2013 Mar 18;54(6):1112–24. doi: 10.1111/epi.12135 (PMC3759702; doi:10.1111/epi.12135)
Supplement: Supplementary file 6 [file epi0054-1112-SD6.pdf]

Physiologic patterns

**Term**	**Definition**
Alpha activity	Rhythmic activity in the alpha frequency range (8-13 Hz), considered normal, but not a part of the background activity / posterior dominant rhythm.
Arousal pattern in children	Prolonged, marked high voltage 4-6/s activity in all leads with some intermixed slower frequencies, in children.
Beta activity	Rhythmic activity in the beta frequency range (14-40 Hz), considered normal, but not a part of the background activity.
Delta activity	Rhythmic activity in the delta frequency range (under 4 Hz), considered normal, but not a part of the background activity.
Diffuse slowing induced by hyperventilation	Bilateral, diffuse slowing during hyperventilation. Recorded in 70% of normal children (3-5 years) and less then 10% of adults. Usually appear in the posterior regions and spread forward in younger age group, whereas they tend to appear in the frontal regions and spread backward in the older age group.
Fast alpha variant rhythm	Characteristic rhythm at 14-20 Hz, detected most prominently over the posterior regions of the head. May alternate or be intermixed with alpha rhythm. Blocked or attenuated by attention, especially visual, and mental effort
Frontal arousal rhythm	Prolonged (up to 20s) rhythmical sharp or spiky activity over the frontal areas (maximum over the frontal midline) seen at arousal from sleep in children with minimal cerebral dysfunction.
K complex	A burst of somewhat variable appearance, consisting most commonly of a high voltage negative slow wave followed by a smaller positive slow wave frequently associated with a

	sleep spindle. Duration > 0.5 s. Amplitude is generally maximal in the frontal vertex. K complexes occur during non-REM sleep, apparently spontaneously, or in response to sudden sensory / auditory stimuli, and are not specific for any individual sensory modality.
Lambda wave	Diphasic sharp transient occurring over occipital regions of the head of waking subjects during visual exploration. The main component is positive relative to other areas. Time-locked to saccadic eye movement. Amplitude varies but is generally below 50 µV.
Photic driving (photic following)	Physiologic response consisting of rhythmic activity elicited over the posterior regions of the head by repetitive photic stimulation at frequencies of about 5-30 Hz. Comments: term should be limited to activity time-locked to the stimulus and of frequency identical or harmonically related to the stimulus frequency. Photic driving should be distinguished from the visual evoked potentials elicited by isolated flashes of light or flashes repeated at very low frequency.
Photomyogenic response	A response to intermittent photic stimulation characterized by the appearance in the record of brief, repetitive muscular artifacts (spikes) over the anterior regions of the head. These often increase gradually in amplitude as stimuli are continued and cease promptly when the stimulus is withdrawn. Comment: this response is frequently associated with flutter of the eyelids and vertical oscillations of the eyeballs and sometimes with discrete jerking mostly involving the musculature of the face and head. (Preferred to synonym: photomyoclonic response).
Positive occipital sharp transient of sleep	Sharp transient maximal over the occipital regions, positive relative to other areas, apparently occurring spontaneously during sleep. May be single or repetitive. Amplitude varies but is generally bellow 50 µV.

Posterior slow waves in youth	Waves in the delta and theta range, of variable form, lasting 0.35 to 0.5 s or longer without any consistent periodicity, found in the range of 6-12 years (occasionally seen in young adults). Alpha waves are almost always intermingled or superimposed. Reactive similar to alpha activity.
Saw-tooth waves	Vertex negative 2-5 Hz waves occurring in series during REM sleep
Saw-toothed bursts	Temporal sharp transients in preterm infants occurring in burst of 3-8 sharp, rhythmic waves at 4-8 Hz, often with high voltage (100-200 µV). Synonym: premature temporal theta.
Sleep spindles	Burst at 11-15 Hz but mostly at 12-14 Hz generally diffuse but of higher voltage over the central regions of the head, occurring during sleep. Amplitude varies but is mostly below 50 µV in the adult.
Slow alpha variant rhythms	Characteristic rhythms mostly at 4-5 Hz, recorded most prominently over the posterior regions of the head. Generally alternate, or are intermixed, with alpha rhythm to which they often are harmonically related. Amplitude varies but is frequently close to 50µV. Blocked or attenuated by attention, especially visual, and mental effort. Comment: slow alpha variant rhythms should be distinguished from posterior slow waves characteristic of children and adolescents and occasionally seen in young adults.
Theta activity	Activity in the theta frequency range, considered normal for the age, taking into account amplitude and distribution too.
Vertex sharp transient	Sharp potential, maximal at the vertex, negative relative to other areas, apparently occurring spontaneously during sleep or in response to a sensory stimulus during sleep or wakefulness. May be single or repetitive. Amplitude varies but rarely exceeds 250 µV. Abbreviation: V wave. Synonym: vertex sharp wave.

Patterns of uncertain significance

Term	Definition
Benign epileptiform transients of sleep (BETS)	Small sharp spikes (SSS) of very short duration and low amplitude, often followed by a small theta wave, occurring in the temporal regions during drowsiness and light sleep. They occur on one or both sides (often asynchronously). The main negative and positive components are of about equally spiky character. Rarely seen in children, they are seen most often in adults and the elderly. Two thirds of the patients have a history of epileptic seizures.
Breach rhythm	Rhythmical activity recorded over cranial bone defects. Usually it is in the 6 to 11/sec range, does not respond to movements.
Fourteen- and 6-Hz positive burst	Burst of arch-shaped waves at 13-17 Hz and/or 5-7-Hz but most commonly at 14 and or 6 Hz seen generally over the posterior temporal and adjacent areas of one or both sides of the head during sleep. The sharp peaks of its component waves are positive with respect to other regions. Amplitude varies but is generally below 75 µV. Comments: (1) best demonstrated by referential recording using contralateral earlobe or other remote, reference electrodes. (2) This pattern has no established clinical significance.
Needle –like occipital spikes of the blind	Spike discharges of a particularly fast and needle-like character develop over the occipital region in most congenitally blind children.<br>Completely disappear during childhood or adolescence.
Rhythmic temporal theta burst of	Characteristic burst of 4-7 Hz waves frequently notched by faster waves, occurring over the temporal regions of the head during drowsiness. Synonym: psychomotor variant pattern. Comment: this is a pattern of drowsiness that is of no clinical significance.

drowsiness (RTTD)	
Rudimentary spike wave complex	Synonym: Pseudo petit mal discharge<br>Paroxysmal discharge that consists of generalized or nearly generalized high voltage 3 to 4/sec waves `with poorly developed spike in the positive trough between the slow waves, occurring in drowsiness only. It is found only in infancy and early childhood when marked hypnagogic rhythmical theta activity is paramount in the drowsy state.
Six Hz spike and slow wave	Spike and slow wave complexes at 4-7Hz, but mostly at 6 Hz occurring generally in brief bursts bilaterally and synchronously, symmetrically or asymmetrically, and either confined to or of larger amplitude over the posterior or anterior regions of the head. The spike has a strong positive component. Amplitude varies but is generally smaller than that of spike-and-slow-wave complexes repeating at slower rates. Comment: this pattern should be distinguished from epileptiform discharges. Synonym: wave and spike phantom.
Slow fused transient	A posterior slow-wave preceded by a sharp-contoured potential that blends together with the ensuing slow wave, in children.
Subclinical rhythmic EEG discharge of adults (SREDA)	A rhythmic pattern seen in the adult age group, mainly in the waking state or drowsiness. It consists of a mixture of frequencies, often predominant in the theta range. The onset may be fairly abrupt with widespread sharp rhythmical theta and occasionally with delta activity. As to the spatial distribution, a maximum of this discharge is usually found over the centroparietal region and especially over the vertex. It may resemble a seizure discharge but is not accompanied by any clinical signs or symptoms.
Temporal	Focal theta and/or delta activity over the temporal regions, especially the left, in persons over

slowing in elderly subjects	the age of 60. Amplitudes are low/ similar to the background activity. Comment: focal temporal theta was found in 20% of people between the ages of 40-59 years, and 40% of people between 60 and 79 years. One third of people older than 60 years had focal temporal delta activity.
Wicket spikes	Spike-like monophasic negative single waves or trains of waves occurring over the temporal regions during drowsiness that have an arcuate or mu-like appearance. These are mainly seen in older individuals and represent a benign variant that is of little clinical significance.
