# Supplementary material for: Standardized Computer-based Organized Reporting of EEG: SCORE
Source: Epilepsia. 2013 Mar 18;54(6):1112–24. doi: 10.1111/epi.12135 (PMC3759702; doi:10.1111/epi.12135)
Supplement: Supplementary file 7 [file epi0054-1112-SD7.pdf]

ARTEFACTS

Term	Definition
50-60 Hz artifact	Monomorphic waveform due to 50 or 60 Hz A/C power supply.
ECG	Far-field potential generated in the heart. The voltage and apparent surface of the artifact vary from derivation to derivation and, consequently, from montage to montage. The artifact is observed best in referential montages using earlobe electrodes A1 and A2. ECG artifact is recognized easily by its rhythmicity/regularity and coincidence with the ECG tracing.
ECG by proxy	ECG from parent or carer
Electrode pops	Are brief discharges with a very steep upslope and shallow fall that occur in all leads which include that electrode
EMG	Myogenic potentials are the most common artifacts. Frontalis and temporalis muscles (ex..: clenching of jaw muscles) are common causes. Generally, the potentials generated in the muscles are of shorter duration than those generated in the brain. The frequency components are usually beyond 30-50 Hz, and the bursts are arrhythmic.
Eye blinks	Fp1/Fp2 become electropositive with eye closure because the cornea is positively charged causing a negative deflection in Fp1/Fp2. If the eye blink is unilateral, consider prosthetic eye. If it is in F8 rather than Fp2 then the electrodes are plugged in wrong.
Eye movements: horizontal	There is an upward deflection in the Fp2-F8 derivation, when the eyes move to the right side. In this case F8 becomes more positive and therefore. When the eyes move to the left, F7 becomes more positive and there is an upward deflection in the Fp1-F7 derivation.
Eye movements: vertical	The EEG shows positive potentials (50–100 µV) with bifrontal distribution, maximum at Fp1 and Fp2, when the eyeball rotated upward. The downward rotation of the eyeball was associated with the negative deflection. The time course of the deflections was similar to the time course of the eyeball movement.
Induction artefacts	Artefacts (usually of high frequency) induced by nearby equipment (like in the intensive care unit).
Slow eye	Slow, rolling eye-movements, seen during drowsiness.

movements (drowsiness)	
Glossokinetic artifact	The tongue functions as a dipole, with the tip negative with respect to the base. The artifact produced by the tongue has a broad potential field that drops from frontal to occipital areas, although it is less steep than that produced by eye movement artifacts. The amplitude of the potentials is greater inferiorly than in parasagittal regions; the frequency is variable but usually in the delta range. Chewing and sucking can produce similar artifacts.
Movement artifact	Large amplitude artifact, with irregular morphology (usually resembling a slow-wave or a wave with complex morphology) seen in one or several channels, due to movement. If the causing movement is repetitive, the artifact might resemble a rhythmic EEG activity.
Photomyogenic response (orbitofrontal photomyoclonus)	A response to intermittent photic stimulation characterized by the appearance in the record of brief, repetitive muscular artifacts (spikes) over the anterior regions of the head. These often increase gradually in amplitude as stimuli are continued and cease promptly when the stimulus is withdrawn. Comment: this response is frequently associated with flutter of the eyelids and vertical oscillations of the eyeballs and sometimes with discrete jerking most involving the musculature of the face and head. A cerebral component of frontal origin may also be present.
Pulse artifact	Occurs when an EEG electrode is placed over a pulsating vessel. The pulsation can cause slow waves that may simulate EEG activity. A direct relationship exists between ECG and the pulse waves (200-300 millisecond delay after ECG equals QRS complex).
Respiration artifacts	Respiration can produce 2 kinds of artifacts. One type is in the form of slow and rhythmic activity, synchronous with the body movements of respiration and mechanically affecting the impedance of (usually) one electrode. The other type can be slow or sharp waves that occur synchronously with inhalation or exhalation and involve those electrodes on which the patient is lying.
Rocking or patting artefact	Quasi-rhythmical artefacts in recordings from infants caused by rocking/ patting
Salt bridge artifact	Typically occurs in 1 channel which may appear isoelectric. Only seen in bipolar montage.
Sweat artifact	Is a low amplitude undulating waveform that is usually > 2 seconds and may appear to be an unstable baseline.
