# Supplementary material for: Standardized Computer-based Organized Reporting of EEG: SCORE
Source: Epilepsia. 2013 Mar 18;54(6):1112–24. doi: 10.1111/epi.12135 (PMC3759702; doi:10.1111/epi.12135)
Supplement: Supplementary file 8 [file epi0054-1112-SD8.pdf]

Specific features of the neonatal EEG

Term	Definition
Active	Behavioural stage for <30 weeks, that describes active behaviour. No differentiation on EEG.
Active sleep	Behavioural stage for >30 weeks, that describes active sleep with rapid eye movement
Alpha	Rhythm with frequency from 8 to 13 Hz.
Beta	Rhythm with frequency from 13 to 40 Hz.
Continuous tracing	(Tracé continu) Physiological for gestational age features; continuous EEG activity with minimal amplitude of 25 µV, lasting at least 1 min. Continuity is present in active in preterm babies ≥28—29 weeks gestational age and in both sleep states in normal full-term babies (≥39 weeks gestational age).
Delta brushes	Consist of high-amplitude slow waves (0.3–1.5 Hz, 50–300 µV) with superimposed bursts of fast activity (> 8 Hz). They are first observed around conceptual age of 2 weeks, have a peak incidence at around 32 weeks, with progressive diminution between 34 and 37 weeks. At first delta brushes are diffuse, then predominant over the temporal and occipital region and are always occipital in the term neonate.
Discontinuous tracing -with physiological bursts	(Tracé discontinu) Characterised by mixed frequency high-voltage bursts separated by periods of very low-voltage background (25 µV). The cumulative duration of interburst intervals corresponds to at least 50% of a 1 min analysis period. Reactivity and liability is present according to CA of the baby.<br>The EEG activity in the bursts is considered normal for age.<br>Normal in preterm infants.
Discontinuous tracing: -with non-physiological bursts	Discontinuous tracing characterised by mixed frequency high-voltage bursts separated by periods of very low-voltage background. The cumulative duration of interburst intervals corresponds to at least 50% of a 1 min analysis period. It may or may not be reactive to external stimuli.<br>The EEG activity in the bursts is considered abnormal for age.
Electrocerebral inactivity	Absence of cortical electric activity.
Frontal sharp transients	Diphasic sharp waves in the frontal area with a voltage of 50–200 µV and a duration of 0.5–0.75 s and appear at about conceptual age of 35 weeks. They can be unilateral or bilateral and may occur at random and in variable frequency.
Gestational age at birth	The time (number of complete weeks) elapsed between the first day of the last normal menstrual period and the day of delivery.
Gestational age at study time	Gestational age corrected at the time of recording the EEG. It is calculated as: gestational age at birth + chronological (postnatal) age at the time of the EEG recording, expressed in weeks.

Induced state	Behavioural stage induced by medication.
Intermediate	Behavioural stage (>30 weeks) between two well defined stages, in which the parameters are disconcordant, also called transitional sleep.
Premature temporal theta	Brief rhythmic bursts of 4–7 Hz, 100–250 µV, sharp theta activity in the temporal regions, sometimes referred to as 'temporal saw-tooth', which are maximal at CA 29 weeks but can be seen less frequently up to conceptual age of 32 weeks.
Quiet	Behavioural stage for <30 weeks, that describes resting behaviour. No differentiation on EEG.
Quiet sleep	Behavioural stage for >30 weeks, that describes sleep without rapid eye movements
Slow anterior dysrhythmia	Bursts of mono- or polyphasic delta waves at 1–3 Hz with a voltage of 50–100 mV over the frontal region appear at conceptual age of 36–37 weeks in active sleep.
STOPs	Sharp Theta rhythm on the Occipital areas of Premature<br>Rhythm with frequency from 4 to 8 Hz, consisting of sharply contoured waves, in the occipital areas.
Suppression-Burst	Pattern characterised by bursts of theta and/or delta waves, at times intermixed with faster waves, and intervening periods of low amplitude (< 20 mV). Comment: an EEG pattern that indicates either severe brain dysfunction or is may be seen with some anaesthetic drugs at certain levels of anaesthesia. It shows no liability or reactivity, and suggests a poor outcome. It differs from discontinuous pattern in preterm infant as it shown little variability, no reactivity or liability and contains no physiological pattern.
Theta	Rhythm with frequency from 4 to 8 Hz.
Tracé alternant	An alternating pattern of non-REM (quiet) sleep seen in term neonates of 36 weeks conceptual age of or older which can persist up to 3–4 weeks after birth in full-term neonates. The pattern is characterised by bursts of predominantly slow waves (1–3 Hz, 50–100 mV) appearing approximately every 4–5 s, and intervening periods of low-voltage activity < 50 mV, 4–7 Hz).
Wakefulness	Behavioural stage for >30 weeks.<br>Active wakefulness characterised by agitation / movement. Quiet wakefulness characterised by bright, wide open eyes, without agitation.
